# p66Shc deletion confers apoptotic resistance to loss of EGFR-ERK signalling in neural stem cells

**DOI:** 10.1038/s41419-025-07778-8

**Published:** 2025-07-01

**Authors:** Andrew M. Powell, Robert C. Cumming, Dean H. Betts

**Affiliations:** 1 Department of Biology, The University of Western Ontario, London, ON Canada; 2 Children’s Health Research Institute, London Health Sciences Centre, London, ON Canada; 3 Department of Physiology and Pharmacology, The University of Western Ontario, London, ON Canada

**Keywords:** Neural stem cells, Apoptosis

## Abstract

Growth factor signalling, through epidermal growth factor (EGF) and its receptor (EGFR), governs neural stem cell (NSC) proliferation, differentiation, and survival. The Src Homology and Collagen (SHC1) adaptor protein mediates EGFR survival-signalling in NSCs via its two shorter isoforms. However, the role of its longest isoform, p66Shc, in NSCs remains unclear. In this study, we investigated the role of p66Shc in NSC apoptosis by generating p66Shc knockout (p66KO) NSCs and assessing their responses to EGF withdrawal, EGFR inhibition, and MEK inhibition. We found that p66KO NSCs resisted apoptosis induced by EGF deprivation and EGFR-ERK pathway inhibition. In contrast, p66KO NSCs maintained their sensitivity to staurosporine, a general apoptosis inducer. Furthermore, p66KO NSCs subjected to prolonged MEK inhibition continued to differentiate into neurons, demonstrating their ability to evade apoptosis and progress through neuronal differentiation. These findings identify p66Shc as a pivotal regulator of NSC apoptosis in response to disrupted EGFR-ERK signalling. The ability of p66KO NSCs to resist apoptosis and differentiate without EGFR-ERK signalling highlights the potential of targeting p66Shc in conditions where growth factor signalling is disrupted, such as neurodegenerative diseases or brain injuries. Additionally, the role of p66Shc in modulating survival pathways may have broader implications for NSC-like cancers, where assessing p66Shc levels could provide prognostic value for the sensitivity of cancers to EGFR- or MEK-inhibition-based chemotherapies.

## Introduction

The apoptosis of neural stem cells (NSCs) is essential for proper brain development, as it maintains a delicate balance between renewing vital progenitor pools and eliminating damaged or surplus cells [1–4]. Disruption of this balance is associated with a wide range of neurological disorders, including developmental neuropathies and brain tumors [5–8]. Central to these processes is growth factor signaling, which governs NSC fate by regulating proliferation, differentiation, and apoptosis. Among the growth factors, epidermal growth factor (EGF) plays a particularly crucial role in these early developmental decisions [9–11].

EGF is routinely added to NSC cultures to support self-renewal and prevent premature differentiation, underscoring its importance in maintaining the balance between survival and cell fate determination [12–15]. EGF exerts its trophic effects by binding to the epidermal growth factor receptor (EGFR). This binding triggers a cascade of intracellular signals, activating several downstream pathways, including the extracellular signal-regulated kinase (ERK) pathway, which is critical for promoting cellular proliferation and survival [16–18]. A key mediator of this signaling is the SHC1 (Src Homology and Collagen) family of adaptor proteins. SHC1 facilitates signal transduction by physically linking activated EGFR to the RAS-MAPK pathway, amplifying ERK signaling [19–22]. As a result, SHC1 is essential for cellular fitness, and its abnormal expression or enhanced activation has been linked to various diseases, most notably cancer [23–29].

Although SHC1 is ubiquitously expressed in most tissues, its expression in the brain is restricted to early developmental stages and is absent in mature neurons [30]. In vivo genetic studies have shown that mutations or deletions of *SHC1* in NSCs cause significant disruptions in brain morphogenesis. Specifically, conditional deletion of *SHC1* in NSCs impairs their proliferation and leads to anatomical disorganization within the NSC niche [31]. Moreover, the inactivation of SHC1 through mutations in its tyrosine phosphorylation sites has been associated with the depletion of the NSC pool and the development of microcephaly, driven by increased NSC apoptosis [32].

The *SHC1* gene encodes three distinct isoforms: p46Shc, p52Shc, and p66Shc, each with unique functions in signal transduction. Notably, p66Shc plays a contrasting role to the shorter isoforms. Unlike p46Shc and p52Shc, which are involved in growth signaling [33–35], p66Shc interacts with mitochondrial components, promoting oxidative stress, mitophagy and apoptosis [33, 36–39]. P66Shc has promoted apoptosis in various NSC-like models, including glioblastomas and immortalized neuronal lines [40–44]. However, its specific role in NSCs remains largely unexplored.

Considering the established role of SHC1 in NSC self-renewal, the antagonistic effect of p66Shc on mitogenic signaling, and its ability to sensitize NSC-like models to neurotoxins, we hypothesized that p66Shc might similarly sensitize NSCs to apoptosis after the withdrawal of mitogenic growth factors by further inhibiting survival pathways. To test this hypothesis, we generated a p66Shc knockout (KO) NSC line and evaluated its sensitivity to EGF withdrawal and targeted inhibition of EGFR and its downstream signaling pathways.

This study demonstrates that deleting *p66Shc* in NSCs confers resistance to apoptosis induced by EGF withdrawal and targeted inhibition of the EGFR-ERK signaling pathway. Our findings highlight the pivotal role of p66Shc in sensitizing NSCs to apoptotic signals in the absence of mitogenic stimuli. This suggests that p66Shc is a crucial regulator of NSC survival, influencing the balance between apoptosis and neuronal differentiation by linking growth factor signaling with apoptotic pathways.

## Materials and methods

Certain aspects of the methodology and their underlying rationale are discussed alongside the results to simplify the presentation of results.

### Cell culture and derivation of NSCs

Wild-type (WT) and p66KO murine NSCs were derived from WT and p66KO embryonic stem cells (mESCs), previously generated using CRISPR-mediated p66Shc deletion [45]. R1 mESC lines were cultured on Nunc cell culture dishes coated with 0.2% (w/v) gelatin in N2B27 media consisting of a 1:1 mixture of KO-DMEM/F12 (Gibco) and Neurobasal (Gibco), supplemented with GlutaMAX (1×, ThermoFisher), 2-Mercaptoethanol (0.1% vol, Millipore Sigma), N2 Supplement (0.5×, ThermoFisher), and B27 Supplement without vitamin A (0.5×, ThermoFisher). The media was further supplemented with CHIR99021 (3 μM, Reagents Direct), PD0325901 (1 μM, Reagents Direct), and mLIF (1000 U/mL, Millipore Sigma).

NSCs were generated by differentiating mESCs using a method adapted from an adherent monolayer differentiation protocol. In brief, mESCs were seeded at 10,000 cells/cm^2^ on 0.2% (w/v) gelatin-coated plates in N2B27 media without LIF or inhibitors. After 6 days, radial neural rosette-like structures were mechanically detached, dissociated using Accutase (ThermoFisher), and seeded on uncoated dishes in N2B27 media supplemented with 20 ng/mL EGF (Peprotech) and 20 ng/mL FGF-2 (Peprotech). After 4 days, the cell aggregates were dissociated and re-seeded onto 0.2% (w/v) gelatin-coated plates in NSC growth media consisting of KO-DMEM/F12 (Gibco), GlutaMAX (1×, ThermoFisher), 2-Mercaptoethanol (0.1% vol, Millipore Sigma), N2 Supplement (1×, ThermoFisher), B27 Supplement without vitamin A (1×, ThermoFisher), EGF (0.2 ng/mL, Peprotech), and FGF-2 (0.2 ng/mL, Peprotech).

The NSC identity of the differentiated cells was confirmed by immunofluorescence (IF) staining for NSC markers Nestin and SOX2 and the absence of the embryonic marker Oct3/4 (Supplementary Fig. S1). Both lines demonstrated the ability to self-renew in EGF/FGF-2-containing media and could differentiate into glial and neuronal lineages (data not shown). NSCs were maintained in NSC growth media at 37 °C in a humidified atmosphere containing 5% CO_2_ on 0.2% (w/v) gelatin-coated plates and passaged two to three times post-thaw before experimentation.

### EGF withdrawal and inhibitor treatments

To assess the effects of EGF withdrawal, NSC growth media were replaced with EGF-free growth media for 24–48 hours. Cell viability was assessed at 48 hours using the trypan blue exclusion assay, while apoptosis was evaluated at 24 hours using Annexin V/PI flow cytometry and IF staining for cleaved caspase-3 and cytochrome C.

### EGFR inhibition

To evaluate the role of EGFR signaling, WT and p66KO NSCs were initially treated with the EGFR-specific inhibitor AG1478 (Selleck) at concentrations of 0.04, 0.4, and 4 μM for 24 hours. Cell viability was assessed using the trypan blue exclusion assay. Subsequent experiments were performed using 2 μM AG1478, with viability and apoptosis assessed at both 24- and 48-hour time points by Annexin V/PI flow cytometry and, at 24 hours, by IF staining for cleaved caspase-3 and cytochrome C.

For immunoblot analysis of EGFR-dependent signaling, NSCs were treated with DMSO vehicle or 2 μM AG1478 for 4 hours, followed by stimulation with 20 nM EGF for 30 minutes prior to protein harvest.

### MEK inhibition

Cells were initially treated with the MEK inhibitor PD0325901 (Reagents Direct) at concentrations of 0.01, 0.1, and 1 μM for 24 hours, and viability was assessed using the trypan blue exclusion assay. Based on these results, all subsequent experiments used 1 μM PD0325901. Apoptosis was evaluated by Annexin V/PI flow cytometry at 24 and 48 hours, and by IF staining for cleaved caspase-3 and cytochrome C at 24 hours.

For immunoblot analysis of ERK1/2-dependent signaling, NSCs were treated with DMSO vehicle or 1 μM PD0325901 for 4 hours, followed by stimulation with 20 nM EGF for 30 minutes. To assess the temporal dynamics of MEK inhibition on ERK signaling and apoptosis-associated proteins, WT and p66KO NSCs were treated with 1 μM PD0325901 and harvested at 0, 4, 14, and 24 hours for immunoblotting.

### PI3K inhibition

The effects of PI3K inhibition were first evaluated by treating NSCs with LY294002 (Selleck) at concentrations of 2, 10, and 50 μM for 24 hours, with viability assessed by trypan blue exclusion assay. For subsequent experiments, 40 μM LY294002 was used. Apoptosis and viability were assessed at 24 and 48 hours by Annexin V/PI flow cytometry.

### General apoptosis induction with staurosporine

To evaluate sensitivity to general apoptosis, WT and p66KO NSCs were treated with staurosporine at concentrations of 0, 100, 200, and 400 nM for 24 hours. To assess apoptotic kinetics, cells were also treated with 400 nM staurosporine for 0, 6, and 24 hours. Apoptotic progression was evaluated by IF staining for cleaved caspase-3 and cytochrome C.

For immunoblot analysis of caspase activation, WT and p66KO NSCs were treated with 400 nM staurosporine or DMSO vehicle for 6 hours and analyzed for caspase-3 and caspase-9 cleavage.

### Mitochondrial stressors

To assess apoptosis induction by mitochondrial ROS stressors, WT and p66KO NSCs were treated with rotenone (Sigma-Aldrich, R8875) or antimycin A (Sigma-Aldrich, A8674). First, a dose-response curve was generated for each compound (rotenone: 0, 0.01, 0.1, 1, and 10 µM; antimycin A: 0, 0.04, 0.2, 1, and 5 µM) over 24 hours, with cell viability evaluated using an MTT assay. Apoptosis was then assessed following treatment with either rotenone (2 µM) or antimycin A (4 µM) for 24 hours. Apoptotic progression was measured by IF staining for cleaved caspase-3 activation and cytochrome C release.

Unless otherwise indicated, inhibitors were initially dissolved in DMSO and further diluted into freshly prepared NSC growth media immediately prior to treatments.

### Treatments with antioxidants and measurement of ROS

N-Acetyl-Cysteine (NAC, Millipore Sigma) was dissolved in sterile PBS, filtered through a 0.22 µm filter, and stored at 4 °C for up to 1 week. MitoTEMPO (mTEMPO, Millipore Sigma) was prepared as a 10 mM stock in sterile water, filtered through a 0.22 µm filter, aliquoted, and stored at −20 °C. Fresh working concentrations of NAC (0.2—2 mM) and MitoTEMPO (1-10 µM) were prepared in cell culture media on the day of the experiment. NSCs were seeded at 20,000 cells/cm² and pre-treated with antioxidants for 1 h prior to treatment with the MEK inhibitor PD0325901 (1 µM, 24 h) without removing the antioxidants.

### Assessing cell death and apoptosis

#### Cell viability assay by trypan blue exclusion assay

Cell viability was assessed using 0.4% Trypan Blue (Thermo Fisher Scientific). WT and p66KO NSCs were harvested, resuspended in media, and mixed 1:1 with Trypan Blue. After 2–3 minutes, viable and non-viable cells were counted using a hemocytometer.

#### MTT assay for cell viability

Cell viability following rotenone and antimycin A treatment was assessed using the MTT assay. WT and p66KO NSCs were plated in 96-well plates at a density of 50,000 cells/cm² (~16,000 cells per well) in NSC growth medium and allowed to adhere overnight. Cells were treated with increasing concentrations of rotenone (0.01–10 μM) or antimycin A (0.04–5 μM) for 24 hours.

At the end of treatment, 100 μL of MTT solution (0.5 mg/mL in PBS) was added to each well, and plates were incubated at 37°C in 5% CO_2_ for 2 hours. Formazan crystals were solubilized by adding 100 μL of DMSO directly to each well, and absorbance was measured at 570 nm using a microplate reader. Absorbance values were normalized to DMSO vehicle controls to determine relative cell viability.

#### Flow cytometry analysis of apoptosis

Apoptosis was analyzed using Annexin V-FITC and propidium iodide (PI) staining following EGF withdrawal, MEK inhibition, EGFR inhibition, or staurosporine treatment for 24 or 48 h. Cells were stained using the Annexin V-FITC Apoptosis Detection Kit (Sigma-Aldrich) and analyzed with a FACSCanto^TM^ flow cytometer (BD Biosciences). FlowJo was used for data analysis. Cells positive for Annexin V+/PI− were considered early apoptotic, Annexin V+/PI+ were late apoptotic or necrotic, and Annexin V−/PI+ were necrotic.

#### Imaging

Phase contrast images of cell morphology were acquired using a Leica DMI 6000 microscope (Leica Microsystems) and Application Suite X software (Leica Microsystems).

#### Apoptotic markers

IF staining was performed to detect cleaved caspase-3 and cytochrome C. NSCs were cultured in laminin-coated 8-well µ-Slides (Ibidi), fixed with 4% paraformaldehyde (Millipore Sigma) for 15 minutes, and blocked with 1% BSA in PBS containing 0.1% Triton X-100 for 1 h. Cells were incubated overnight at 4°C with primary antibodies: cleaved caspase-3 (1:400, Cell Signaling, 9664) and cytochrome C (1:400, Invitrogen, 338200), followed by appropriate secondary antibodies. Nuclei were counterstained with Hoechst 33342. Fluorescence images were captured using a Nikon Eclipse Ti2E microscope and analyzed with NIS Elements software.

#### Cell identity markers

IF staining was performed to detect Nestin (1:400, Abcam, ab6142) and Doublecortin (DCX) (1:400, Abcam, ab18723). Cells were categorized as uncommitted NSCs (Nestin+/DCX−), neuronally committed cells (DCX + ), or unknown identity (Nestin-/DCX-). For extended MEK inhibition, p66KO NSCs were treated with 1 µM PD0325901 for 7 days, with daily media replacements, and stained for Nestin, GFAP (Abcam, ab7260), and βIII-tubulin (Abcam, ab78078) to assess differentiation.

#### Measurement of reactive oxygen species (ROS) and oxidative stress

Mitochondrial superoxide was detected using MitoSOX Red (Thermo Fisher Scientific), and oxidative stress and mitochondrial membrane potential were measured using CellROX Green and MitoTracker Red (Thermo Fisher Scientific), respectively. NSCs were seeded into laminin-coated 8-well Ibidi plates (Milipore Sigma, L2020) at 30,000 cells/cm^2^, treated with either 1 µM PD0325901 or DMSO for 8 h, and incubated with 2 µM MitoSOX Red or a combination of 500 nM CellROX Green and 20 nM MitoTracker Red for 30 minutes at 37 °C, 5% CO_2_. Nuclei were counterstained with NucBlue Live Stain (Thermo Fisher Scientific), and cells were imaged using a Nikon Eclipse Ti2E fluorescence microscope. CellROX Green to MitoTracker Red fluorescence ratio was calculated per cell to assess oxidative stress relative to mitochondrial metabolism.

#### Immunoblot analysis

NSCs were lysed using RIPA buffer (Millipore Sigma) containing protease and phosphatase inhibitors (Millipore Sigma). Protein concentrations were determined using the BCA protein assay (Thermo Fisher Scientific), and proteins were resolved by SDS-PAGE and transferred to PVDF membranes. Immunoblotting was performed to detect P-ERK1/2 (T202/Y204), ERK, P-AKT (S473), AKT, P-STAT3 (S727), P-STAT (Y705), P-SHC (Y239/240), P-SHC (Y317), SHC, cleaved caspase-3, procaspase-3, caspase-9, BAX, P-BCL2 (S84), P-BCL2 (S70) and BCL2. Membranes were probed with primary antibodies, followed by horseradish peroxidase-conjugated secondary antibodies, and visualized using enhanced chemiluminescence (Immobilon® Classico Western HRP Substrate, Millipore Sigma). Densitometric analysis was performed using ImageLab (Bio-Rad). See Supplementary Table 1 for the complete list of antibodies used and Original Data for full-length blots.

#### RNA extraction and quantitative PCR (qPCR)

Cells were scraped and lysed in TRIzol reagent (Invitrogen, ThermoFisher) and stored at –80 °C until RNA extraction. Total RNA was extracted using chloroform phase separation, followed by purification with the RNeasy Mini Kit (Qiagen) according to the manufacturer’s instructions. Genomic DNA was removed using the RNase-Free DNase Set (Qiagen). First-strand cDNA synthesis was performed using SuperScript III Reverse Transcriptase (Invitrogen, ThermoFisher). Quantitative PCR was carried out using the SensiFAST™ SYBR No-ROX Kit (FroggaBio) on a CFX384 real-time thermal cycler (Bio-Rad). Relative gene expression was calculated using the ΔΔCt method, normalized to Gapdh and β-Actin housekeeping genes. Primer sequences are provided in Supplementary Table 2.

### Statistical analysis

All experiments were conducted in biological triplicate from independent repeats. Data are expressed as mean ± standard error of the mean (SEM). Statistical analysis was performed using GraphPad Prism software, with comparisons made using unpaired Student’s *t* test or two-way ANOVA with Sidak post-hoc tests for normally distributed data.

## Results

### p66Shc deletion protects NSCs from EGF deprivation-induced apoptosis

**Fig. 1:**
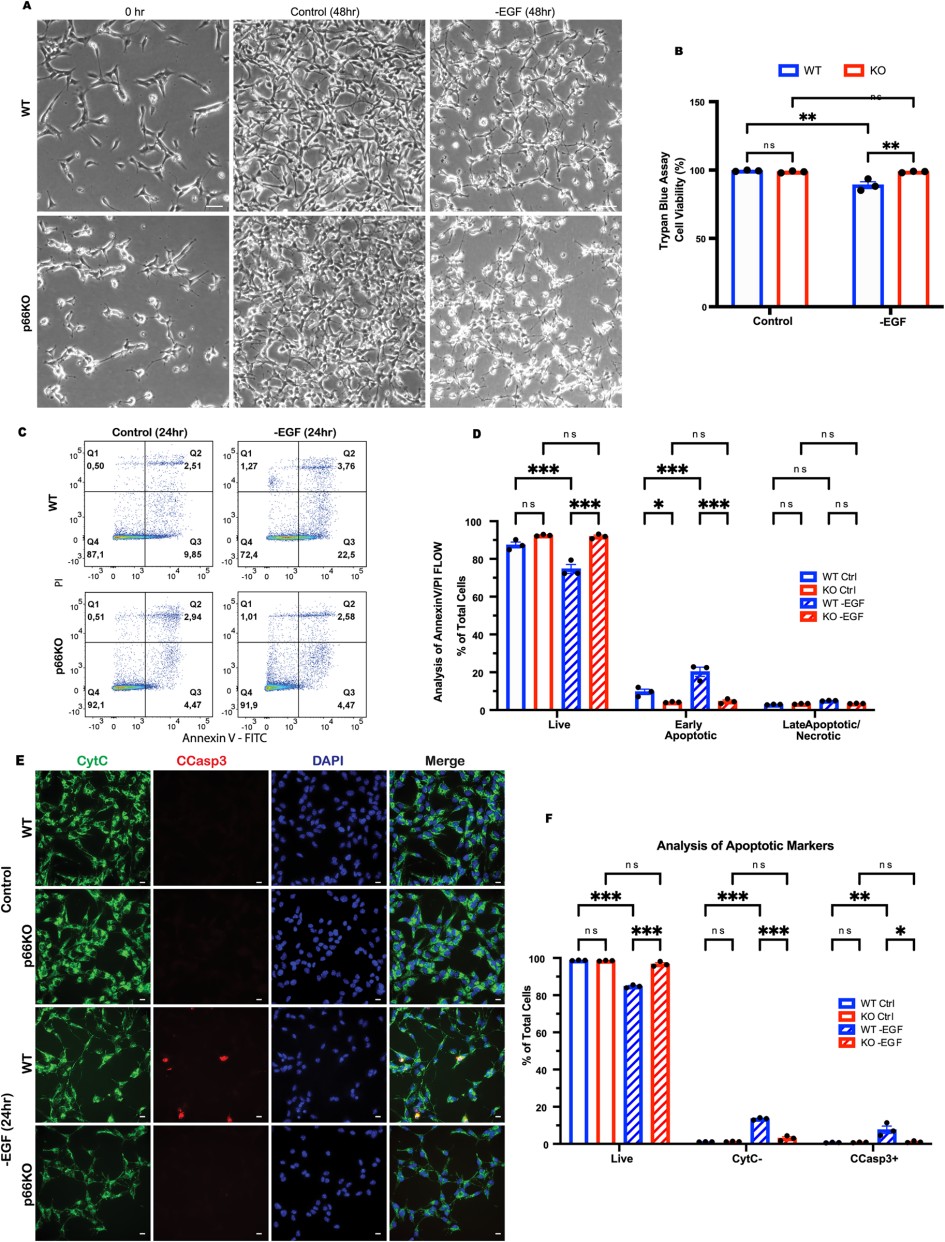
Deletion of p66Shc prevents apoptosis of NSCs following EGF withdrawal. **A** Representative phase contrast images of WT and p66KO NSCs after 48 h in NSC growth media (control), or after 48 h in NSC growth media without EGF. Scale = 20 μm. **B** Effect of 48 h of EGF withdrawal on the percentage of live cells in WT and p66KO NSCs compared to in growth media (control), assessed by trypan blue exclusion assay. Showing mean ± SEM, *n* = 3 independent experiments. **C**, **D** Flow cytometry analysis of annexin V and PI staining in WT and p66KO NSCs treated with (control) or without EGF (-EGF) for 24 h. Representative flow cytometry plots (**C**), and quantification of live (Q1), early apoptotic (Q2), and late apoptotic/necrotic cells (Q3/4) (**D**). Showing mean ± SEM, *n* = 3 independent experiments. **E**, **F** Immunofluorescence (IF) analysis of native cytochrome C (CytC) staining and cleaved caspase-3 (CCasp3) staining, representative image of WT and p66KO NSCs after 24 hr in growth media (Control) or 24 hours in growth media without EGF (-EGF) (**E**) Scale = 10 μm. Quantification of cells negative for CytC, positive for CCasp3, and live cells (positive for CytC, negative for CCasp3) (**F**); mean ± SEM, *n* = 3 independent experiments, each with at least three fields of view. Statistics were obtained using two-way ANOVA: ns, *p* $\ge$0.05; *p < 0.05; **p < 0.01; ***p < 0.001.

To determine how p66Shc deletion affects NSC survival during EGF deprivation, we withdrew EGF from the culture media and assessed cell viability after 48 h. Both WT and p66KO NSCs exhibited reduced proliferation and morphological changes, such as spiny extensions, which are consistent with early stages of differentiation (Fig. 1A). After 48 h without EGF, the WT NSCs began to die. At the same time, the p66KO NSCs maintained their viability (Fig. 1B).

To determine the type of cell death induced by EGF withdrawal, we performed Annexin V/PI flow cytometry and IF staining for apoptosis markers cleaved caspase-3 and cytochrome C. After 24 h without EGF, WT NSCs exhibited signs of early apoptosis, indicated by Annexin V positivity (Fig. 1C, D), while p66KO NSCs showed no significant apoptotic changes. IF staining in WT NSCs confirmed activation of the intrinsic apoptotic pathway through increased cleaved caspase-3 and cytochrome C release (Fig. 1E, F). In contrast, p66KO NSCs did not display elevated levels of these apoptotic markers, suggesting that p66Shc deletion protects NSCs from EGF withdrawal-induced apoptosis by preventing the initiation of apoptosis.

### p66Shc KO NSCs are resistant to EGFR inhibition-induced apoptosis

Previous studies have reported that p66Shc influences both apoptosis and EGFR signaling [33, 46]. Therefore, we hypothesized that deletion of p66Shc might affect apoptosis by modulating EGFR-mediated survival pathways. To test this hypothesis, we treated WT and p66KO NSCs with the EGFR-specific inhibitor AG1478 (EGFRi).

**Fig. 2:**
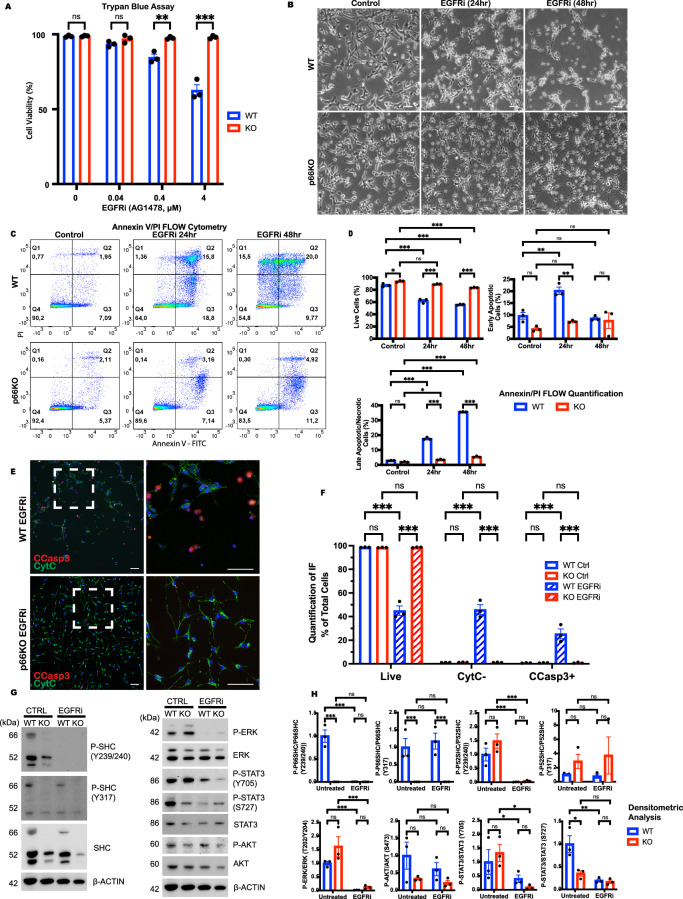
Loss of p66Shc protects against EGFR inhibition-mediated apoptosis. **A** Effect of AG1478 concentration on cell viability after 24 h treatment in WT and p66KO NSCs in NSC growth media, assessed by trypan blue exclusion assay; mean ± SEM, *n* = 3 independent experiments. **B** Representative phase contrast images of WT and p66KO NSCs for the following conditions: NSC growth media 24 h (Control) or treated with 2 μM AG1478 (EGFRi) for 24 h and 48 h. Scale = 20 μm. **C**, **D** Flow cytometry analysis of annexin V and PI staining in WT and p66KO NSCs after 48 h in NSC growth media (Control) or treated with 2 μM AG1478 for 24 h and 48 h. Representative flow cytometry plots (**C**), and quantification of live (Q1), early apoptotic (Q2), and late apoptotic/necrotic cells (Q3/4) (**D**); mean ± SEM, *n* = 3 independent experiments. **E**, **F** IF analysis of native CytC and CCasp3 staining in WT and p66KO NSCs treated with 2 μM AG1478, using ×20 (left) and ×60 (right) magnification. The hatched box indicates the magnified area. Scale = 50 μm (**E**). Quantification of cells negative for CytC, positive for CCasp3, and live cells (positive for CytC, negative for CCasp3) (**F**); mean ± SEM, *n* = 3 independent experiments, each with at least three fields of view. **G**, **H** Western blot analysis of EGFR-mediated signaling proteins including AKT, STAT3, ERK, and SHC in WT and p66KO NSCs treated with DMSO vehicle (CTRL) or 2 μM AG1478 for 4 h, followed by 20 nM EGF stimulation for 30 minutes. Densitometric analysis of the western blots (**H**); mean ± SEM, *n* = 3 independent experiments. Statistics were obtained using two-way ANOVA: ns, *p*$\ge$0.05; *p < 0.05; **p < 0.01; ***p < 0.001.

Treatment with AG1478 resulted in a dose-dependent decrease in the survival of the WT NSCs (Fig. 2A), including characteristic morphological changes such as cell rounding, detachment, and the formation of apoptotic bodies (Fig. 2B). In contrast, p66KO NSCs maintained their viability, attachment, and extended neurite-like projections as previously observed during EGF withdrawal.

We then used Annexin V/PI flow cytometry to characterize apoptosis after treatment with AG1478 (Fig. 2C, D). By 24 h, WT NSCs showed a significant increase in early apoptotic cells, which advanced to late apoptosis/necrosis by 48 h. The p66KO NSCs, however, showed minimal early apoptosis at 24 h, which remained significantly lower than in WT at 48 h. Immunofluorescent staining of WT NSCs showed a marked increase in cytochrome C release and Cleaved Caspase-3 staining (Fig. 2E, F), confirming mitochondrial pathway involvement. In contrast, the p66KO NSCs exhibited negligible levels of these apoptotic markers.

To further investigate the molecular mechanisms underlying the altered sensitivity of p66KO NSCs, we performed immunoblot analysis of EGFR downstream effectors, including P-ERK, P-AKT, and P-STAT3 (Fig. 2G, H). Treatment with AG1478 resulted in the attenuation of SHC phosphorylation at Y239/240 in both WT and KO cell lines, while Y317 phosphorylation remained unaffected, indicating that EGFR may be specifically mediating the phosphorylation of the SHC(Y239/240) residue. Simultaneously, we observed a significant decrease in the phosphorylation of ERK and STAT3 in both WT and p66KO NSCs following EGFR inhibition. WT NSCs showed a more pronounced ERK activity decrease than p66KO NSCs. Though the p66KO NSCs had similar or lower levels of P-STAT3 and P-AKT, the partial retention of ERK signaling in p66KO NSCs may explain their enhanced survival under EGFR inhibition.

### p66Shc KO NSCs resist apoptosis induced by MEK inhibition

**Fig. 3:**
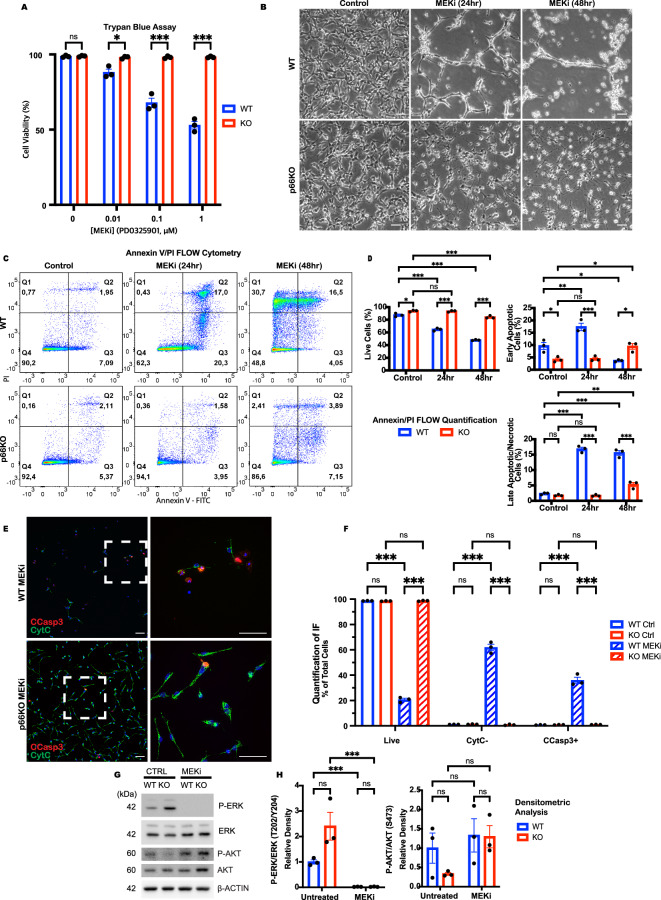
Loss of p66Shc protects against MEK inhibition-mediated apoptosis. **A** Effect of pd032901 concentration on cell viability after 24 h treatment in WT and p66KO NSCs, assessed by trypan blue exclusion assay; mean ± SEM, *n* = 3 independent experiments. **B** Representative phase contrast images of WT and p66KO NSCs for the following conditions: NSC growth media 24 h (Control) or treated with 1 μM pd032901 (MEKi) for 24 h and 48 h. Scale = 20 μm. **C**, **D** Flow cytometry analysis of annexin V and PI staining in WT and p66KO NSCs after 48 h in NSC growth media (control) or treated with 1 μM pd032901 for 24 h and 48 h. Representative flow cytometry plots (**C**), and quantification of live (Q1), early apoptotic (Q2), and late apoptotic/necrotic cells (Q3/4) (**D**); mean ± SEM, *n* = 3 independent experiments. **E**, **F** IF analysis of CytC release and CCasp3 staining in WT and p66KO NSCs treated with 1 μM pd032901, using ×20 (left) and ×60 (right) magnification. The hatched box indicates the magnified area. Scale = 50μm. Quantification of cells negative for CytC, positive for CCasp3, and live cells (positive for CytC, negative for CCasp3) (**F**); mean ± SEM, *n* = 3 independent experiments, each with at least three fields of view. **G**, **H** Western blot analysis of ERK and AKT signaling proteins in WT and p66KO NSCs treated with either DMSO vehicle (CTRL) or 1 μM pd032901 for 4 h, followed by 20 nM EGF stimulation for 30 minutes. Representative blots (**G**), and their densitometric analysis (**H**); mean ± SEM, *n* = 3 independent experiments. Statistics were obtained using two-way ANOVA: ns, *p* ≥ 0.05; *p < 0.05; **p < 0.01; ***p < 0.001.

The pronounced effect of AG1478 on ERK signalling prompted us to investigate whether the suppression of ERK activity might be the primary driver of apoptosis of the WT NSCs, and, conversely, whether its retention was responsible for the survival of the p66KO NSCS. We treated the NSCs with the MEK inhibitor PD0325901 to test these hypotheses. WT NSCs exhibited a dose-dependent decrease in survival (Fig. 3A), characterized by cell rounding, detachment, and the formation of apoptotic bodies (Fig. 3B). In contrast, p66KO NSCs were resistant to MEK inhibition, displaying neurite-like extensions without morphological signs of apoptosis.

To characterize the apoptotic response, we performed Annexin V/PI flow cytometry on WT and p66KO NSCs after MEK inhibition (Fig. 3C, D). WT NSCs showed a significant increase in early apoptosis at 24 h, progressing to late apoptosis and necrosis by 48 h. In contrast, p66KO NSCs exhibited negligible changes after 24 h, and only marginal cell death after 48 h. Immunfluorescent staining confirmed that MEK inhibition-induced apoptosis in WT NSCs (Fig. 3E, F), as evidenced by increased staining for cleaved caspase-3 and cytochrome C release. Notably, the p66KO NSCs, again, showed no significant apoptotic activity.

Immunoblot analysis showed that MEK inhibition blocked ERK phosphorylation in both WT and p66KO NSCs (Fig. 3G, H), indicating that p66KO resistance to apoptosis is not due to retained ERK signaling. To independently confirm ERK pathway inhibition, we measured the expression of the ERK-responsive genes CCND1, DUSP6, EGR1, ETV4, and SPRY2 following MEK inhibitor treatment (0, 2, and 6 h). The expression of all five genes was markedly reduced in both WT and p66KO NSCs, which was consistent with the effective suppression of ERK signaling (Supplementary Fig. S2).

These results further support our conclusion that differential apoptosis between genotypes is not due to differences in ERK pathway activity. MEK inhibition-induced loss of ERK signalling mirrored the effects of EGFR inhibition, triggering apoptosis in WT NSCs but not in p66KO NSCs. This reinforces the idea that ERK suppression is a critical driver of apoptosis in WT NSCs. It also suggests that p66Shc plays a distinct role in apoptosis, independent of its impact on survival signaling pathways.

### p66Shc KO NSCs retain a functional apoptotic response to staurosporine

To investigate whether the absence of p66Shc impacts the broader apoptotic machinery, we assessed the response of NSCs to staurosporine, a potent inducer of apoptosis. This experiment allowed us to determine whether the resistance observed in p66KO NSCs to EGFR and MEK inhibition was a specific response or indicative of a more generalized resistance to apoptosis.

**Fig. 4:**
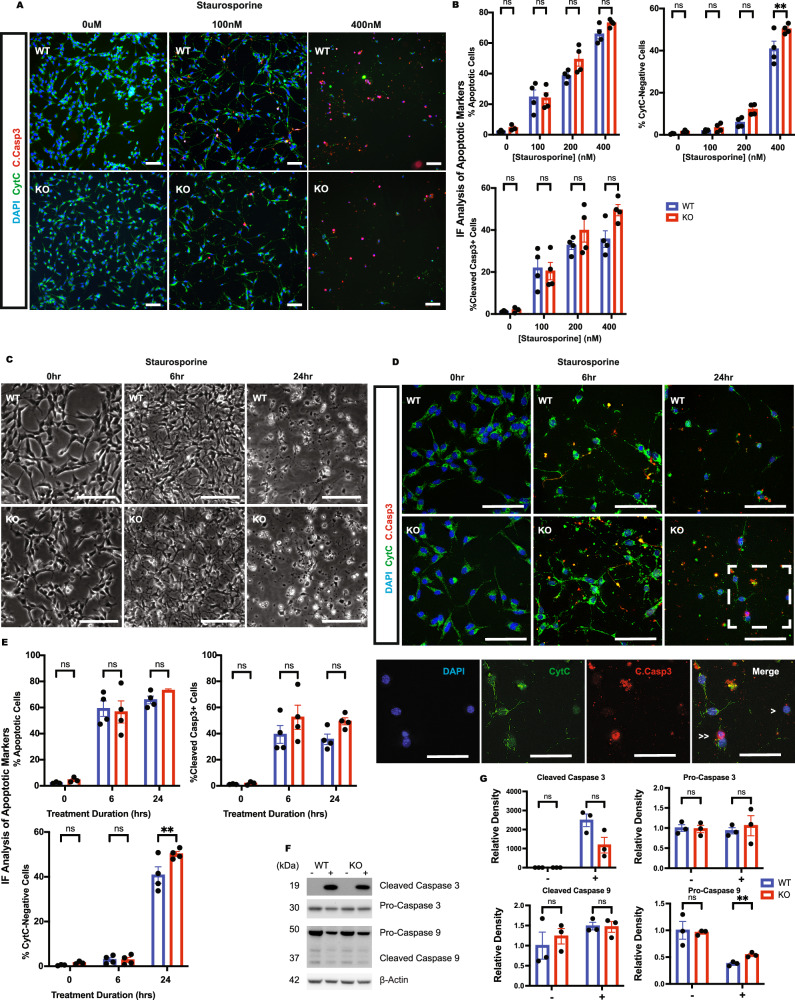
Loss of p66Shc does not protect against staurosporine-mediated apoptosis. **A**, **B** Effect of staurosporine concentration on CytC release and CCasp3, assessed by IF after treatment for 24 h. Scale = 50 μm. Quantification of percentage of apoptotic cells (negative for CytC staining or positive for CCasp3), percent of cell population positive for CytC (CytC-negative) and percent of cell population positive for cleaved caspase-3 (Cleaved Casp3 + ). mean ± SEM, *n* = 3 independent experiments, each with at least three fields of view. **C**–**E** Effect 400 nM staurosporine on apoptosis of WT and p66KO NSCs over 24 h time-course. Representative phase contrast images showing morphological changes to WT and p66KO NSCs. Scale = 50 μm (**C**). IF images showing representative images of WT and p66KO NSCs stained for native CytC and CCasp3 after staurosporine treatment. The hatched box indicates the magnified area, showing p66KO cells undergoing release of CytC (>), and caspase-3 activity and chromatin condensation (>>). Scale = 50 μm (**E**). Quantification of the percentage of apoptotic cells (negative for CytC staining or positive for CCasp3), percent of cell population positive for CytC (CytC-negative) and percent of cell population positive for cleaved caspase-3 (Cleaved Casp3+). mean ± SEM, *n* = 3 independent experiments, each with at least three fields of view. **F**,**G** Western blot analysis of cleaved and procaspase-9 and -3 in WT and p66KO NSCs treated with 400 nM staurosporine for 6 h. Representative blots (**F**) and densitometric analysis (**G**); mean ± SEM, *n* = 3 independent experiments. Statistics were obtained using two-way ANOVA: ns, *p* ≥ 0.05; *p < 0.05; **p < 0.01; ***p < 0.001.

WT and p66KO NSCs were treated with increasing concentrations of staurosporine, and we quantified cytochrome C release and cleaved caspase-3 activation by IF (Fig. 4A, B). Both cell types showed dose-dependent activation of apoptotic markers, with similar levels of apoptosis at each staurosporine concentration.

A time-course analysis revealed that both WT and p66KO NSCs displayed typical apoptotic features, including apoptotic bodies by 24 h (Fig. 4C). IF confirmed nuclear condensation, cytochrome C release, and caspase-3 activation in both groups, with similar rates of apoptotic progression (Fig. 4D, E).

To further assess caspase activation, we examined pro- and cleaved caspase-9 and caspase-3 levels by immunoblotting after 6 h of staurosporine treatment (Fig. 4F, G). Both genotypes showed a marked decrease in procaspase-9, indicating activation of the intrinsic pathway. Although we did not observe an increase in cleaved caspase-9 at this time point, this may be due to sampling just after the transient peak of caspase-9 cleavage. We also observed robust accumulation of cleaved caspase-3 in both WT and KO NSCs, further supporting activation of the apoptotic cascade.

Together, these findings indicate that p66KO NSCs retain a functional intrinsic apoptotic response and that their resistance to MEK inhibition reflects a stimulus-specific defect rather than a general impairment in apoptotic signaling.

### p66Shc KO NSCs Resist MEK Inhibition Independently of PI3K-AKT Signalling

**Fig. 5:**
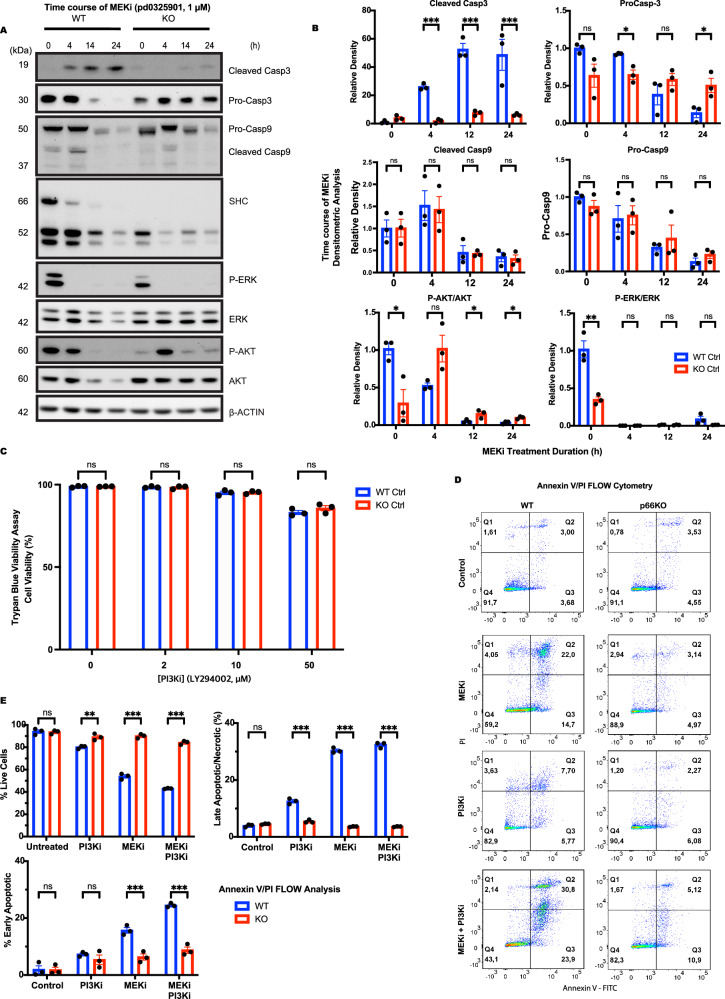
Loss of p66Shc protects against MEK inhibition-mediated apoptosis without enhancing pro-survival ERK or AKT signalling. **A**, **B** Western blot analysis of cleaved caspase-3, procaspase-3, cleaved caspase-9, procaspase-9, P-AKT, AKT, P-ERK, ERK, and SHC in WT and p66KO NSCs treated with 1 μM PD0325901 over a 24 h time course. Representative blots (**A**) and densitometric analysis (**B**); mean ± SEM, *n* = 3 independent experiments. **C** Effect of PI3K inhibitor LY294002 concentration on WT and p66KO NSC viability after 24 h, assessed by trypan blue exclusion assay; mean ± SEM, *n* = 3 independent experiments. **D**, **E** Flow cytometry analysis of Annexin V and PI staining in WT and p66KO NSCs treated with DMSO, 1 μM PD0325901, 40 μM LY294002, or both inhibitors for 24 h. Representative flow plots (**D**) and quantification of live (Q1), early apoptotic (Q2), and late apoptotic/necrotic cells (Q3/4) (**E**); mean ± SEM, *n* = 3 independent experiments. Statistics were obtained using two-way ANOVA: ns, *p* ≥ 0.05; *p < 0.05; **p < 0.01; ***p < 0.001.

While initial ERK signaling loss was observed within 2 h of MEK inhibition, the long-term dynamics of ERK and AKT remained unclear. We hypothesized that p66KO NSCs might restore ERK signaling later or compensate via alternative survival pathways like AKT. To test this, we performed a 24-h time-course analysis, treating WT and p66KO NSCs with the MEK inhibitor PD0325901 and assessing ERK and AKT phosphorylation alongside caspase-9 and casapase-3 cleavage (Fig. 5A, B). MEK inhibition induced a transient increase in cleaved caspase-9 at 4 hours and a progressive reduction in procaspase-9 over 24 hours in both WT and p66KO NSCs, indicating that upstream intrinsic apoptotic signaling is activated in both genotypes. In WT NSCs, this was followed by caspase-3 activation, as evidenced by decreased procaspase-3 and accumulation of cleaved caspase-3. In contrast, p66KO NSCs maintained procaspase-3 levels with minimal cleaved caspase-3 detected across the 24-hour treatment period. Both cell types maintained suppressed P-ERK levels throughout the time-course, confirming that p66KO survival is not due to restored ERK signaling. MEK inhibition also reduced P-AKT in both genotypes; however, p66KO NSCs exhibited a distinct increase in P-AKT at later time points, suggesting that AKT activation may contribute to their resistance.

To explore whether BCL2 signaling plays a role in p66KO resistance, we also examined total BCL2, BAX, and BCL2 phosphorylation at ERK-regulated sites S70 and S84. MEK inhibition led to a similar reduction in BCL2 phosphorylation across genotypes, with no differences in total BCL2, BAX, or BAX/BCL2 ratios (Supplementary Fig. S3). These findings indicate that BCL2 signaling is equally affected in WT and p66KO NSCs, suggesting that the p66KO survival advantage likely depends on mechanisms further downstream of the intrinsic apoptotic cascade.

We treated both NSC types with the PI3K inhibitor LY294002 to evaluate PI3K-AKT’s role in MEKi-mediated apoptosis (Fig. 5C). Both WT and KO NSCs showed low sensitivity to PI3K inhibition, indicating that blocking PI3K-AKT signaling alone did not significantly induce apoptosis or affect survival differently between the groups.

Further, we treated NSCs with a combination of MEK and PI3K inhibitors and assessed apoptosis via Annexin V/propidium iodide flow cytometry (Fig. 5D, E). Even with combined inhibition, p66KO NSCs maintained significantly higher survival rates than WT NSCs, indicating that PI3K inhibition did not eliminate their resistance to MEK inhibition.

These findings suggest that while AKT signaling is modulated by MEK inhibition, the primary driver of apoptosis in WT NSCs is the loss of ERK signaling. The resistance of p66KO NSCs to MEK inhibition is not mediated by compensatory PI3K-AKT signaling, pointing to alternative mechanisms underlying this resistance. Notably, although both genotypes initiate upstream intrinsic apoptotic signalling—as indicated by caspase-9 activation—only WT NSCs proceed to caspase-3 cleavage.

### Antioxidants do not rescue NSCs from MEKi-induced apoptosis

Though p66Shc is linked to cell stress and apoptosis, its exact mechanisms remain unclear. Some studies suggest it promotes apoptosis via ERK and PI3K pathways, while others propose it acts as a mitochondrial redox modulator, increasing ROS. To investigate whether p66Shc mediates MEK inhibition-induced apoptosis through ROS, we measured superoxide production using MitoSOX Red and assessed oxidative stress with CellROX in WT p66KO NSCs after PD0325901 treatment.

**Fig. 6:**
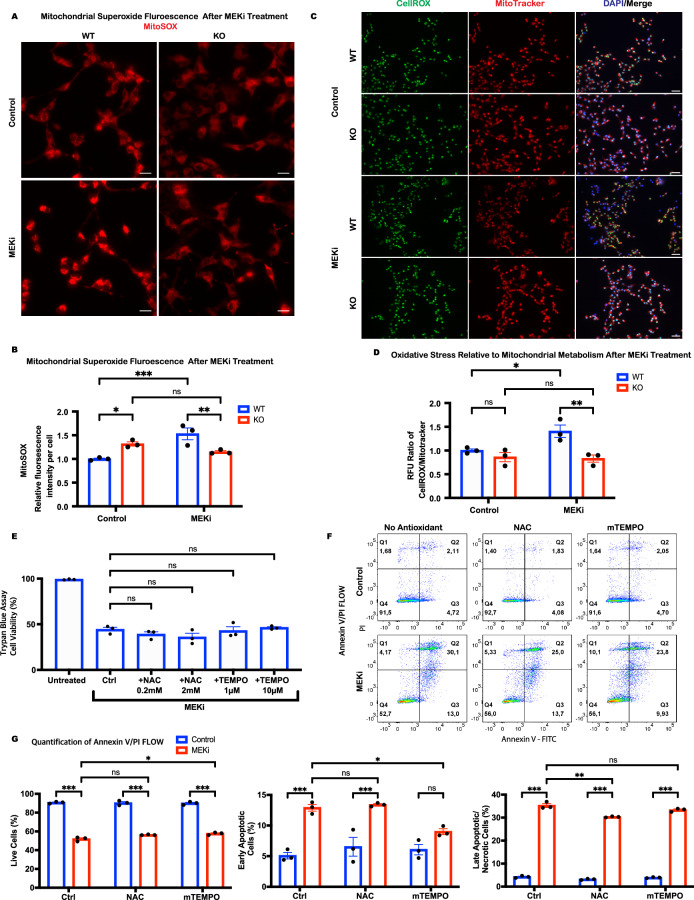
Resistance of p66KO NSCs to MEK inhibition is not mediated by decreased ROS production. **A**, **B** Representative IF images of MitoSOX Red staining in WT and p66KO NSCs following either DMSO vehicle (control) or 1 μM pd032901 treatment in NSC growth media to assess mitochondrial superoxide production (**A**). Quantification of MitoSOX Red fluorescence intensity per cell; mean ± SEM, *n* = 3 independent experiments, each with at least three fields of view. **C**, **D** Representative IF images of CellROX and Mitotracker Red staining in WT and p66KO NSCs following 1 μM pd032901 treatment to assess ROS production and mitochondrial metabolism (**D**). Quantification of fluorescence using the ratio of CellROX/MitoTracker to assess the relative ROS production per metabolic unit; mean ± SEM, *n* = 3 independent experiments, each with at least three fields of view. **E** Effect of antioxidant N-acetyl cysteine (NAC) and mitoTEMPO (mTEMPO) concentrations on WT NSC viability during 1 μM pd032901 treatment, assessed by trypan blue exclusion assay; mean ± SEM, *n* = 3 independent experiments. **F**, **G** Flow cytometry analysis of annexin V and PI staining in WT NSCs following simultaneous treatment with 1 μM pd032901 and either 1 mM NAC or 5 μM mitoTEMPO for 24 h. Representative flow cytometry plots (**F**). Quantification of live (Q1), early apoptotic (Q2), and late apoptotic/necrotic cells (Q3/4) (**G**); mean ± SEM, *n* = 3 independent experiments. Statistics were obtained using two-way ANOVA: ns, *p* ≥ 0.05; *p < 0.05; **p < 0.01; ***p < 0.001.

MEK inhibition significantly increased superoxide production and oxidative stress in WT NSCs but not in p66KO NSCs (Fig. 6A–D), indicating that p66Shc is necessary for these effects. We treated WT NSCs with antioxidants N-acetyl cysteine (NAC) and MitoTEMPO to determine if ROS plays a key role in apoptosis. Neither treatment significantly enhanced NSC survival (Fig. 6F). However, Annexin V/PI flow cytometry revealed that MitoTEMPO significantly reduced early apoptosis in WT NSCs (Fig. 6G, H). Despite this reduction, overall survival remained significantly lower than in untreated controls.

To further explore whether p66Shc modulates apoptosis through its role in mitochondrial ROS signaling, we assessed the response of WT and p66KO NSCs to rotenone and antimycin A, two mitochondrial complex inhibitors that generate ROS through distinct mechanisms. p66KO NSCs exhibited partial resistance to rotenone, but not antimycin A, as measured by MTT viability and apoptotic markers (Supplementary Fig. S4). These findings support a role for p66Shc in regulating sensitivity to specific mitochondrial ROS-dependent stressors, though its effect appears context-dependent and less pronounced than in the case of MEK inhibition.

These findings suggest that p66Shc contributes to ROS production and oxidative stress following MEK inhibition, and that ROS may play a modulatory role in apoptosis initiation. However, the limited protective effect of antioxidant treatment and the incomplete resistance of p66KO NSCs to mitochondrial ROS stressors may indicate that ROS is not the sole driver of apoptosis in this context.

### p66Shc KO NSCs exhibit apoptotic resistance and neuronal differentiation under sustained MEK inhibition

Cell identity influences sensitivity to stressors—neurons are less dependent on EGF for survival than NSCs [47, 48]. Consequently, the observed differences in apoptosis between WT and p66KO NSCs may result from variations in cell identity, differentiation capacity, or fate-specific resistance to the loss of EGFR-ERK signaling.

**Fig. 7:**
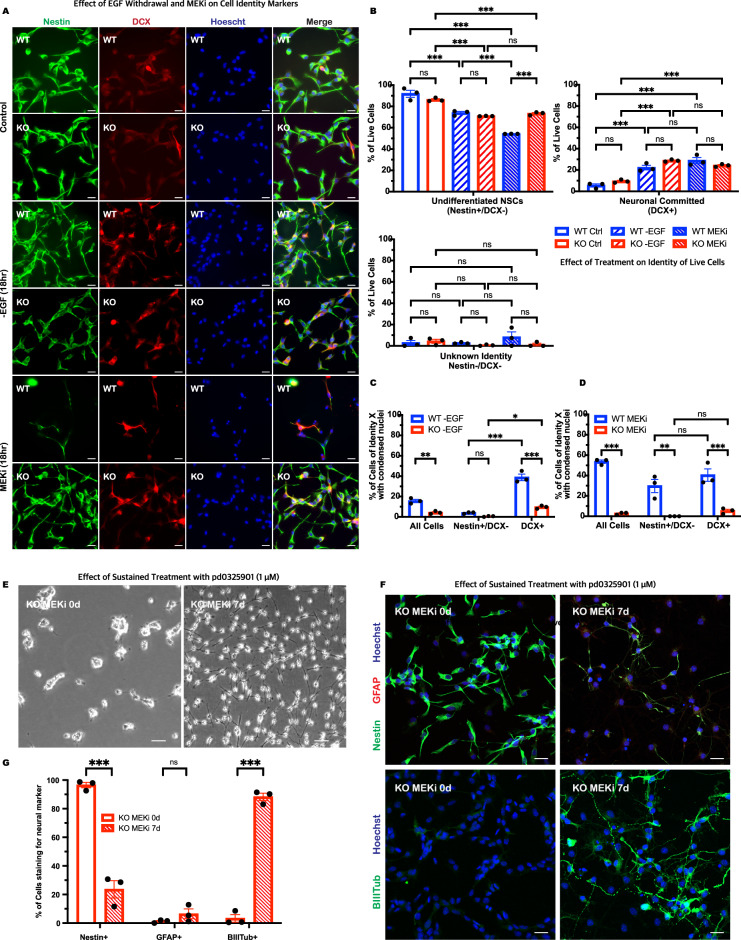
Loss of p66Shc permits neuronal differentiation of NSCs during continuous MEK inhibition without influencing uncommitted or neuronal identities. **A**–**D** Representative IF images of WT and p66KO NSCs after EGF withdrawal (-EGF) or 18 h treatment with 1 μM pd032901 (MEKi), stained for NSC marker Nestin and neuronal commitment marker DCX (**A**). Quantification of live cells positive for each marker, assessing percentage of cells that are uncommitted NSCs (Nestin + /DCX−), neuronally committed (DCX+) and of unknown identity (Nestin-−/DCX−) (**B**). Percentage of condensed nuclei of each identity relative to the total number of cells of that identity to assess the sensitivity of each neural identity to EGF withdrawal (**C**) and MEK inhibition (**D**). Mean ± SEM, *n* = 3 independent experiments, each with at least three fields of view. **E**, **G** Effect of 7 days of continuous MEK inhibition on p66KO NSC identity. Representative phase contrast images of p66KO NSCs after 7-day treatment with 1 μM pd032901 (**E**). IF images of p66KO NSCs after 7-day treatment, stained for NSC marker Nestin, glial marker GFAP, and neuronal marker BIII-Tubulin (**F**). Quantification of cells positive for each marker, assessing percent of the cell population positive for Nestin, GFAP and BIII-Tubulin (**G**). Mean ± SEM, *n* = 3 independent experiments, each with at least three fields of view. Statistics were obtained using two-way ANOVA: ns, *p* ≥ 0.05; *p < 0.05; **p < 0.01; ***p < 0.001.

To determine whether the resistance of p66KO NSCs to apoptosis is related to differences in cell identity or differentiation capacity, we analyzed the expression of the identity markers Nestin and doublecortin before and after EGF withdrawal or MEK inhibition (Fig. 7A, B). Prior to treatment, there were no significant differences in the identity profiles of WT and p66KO NSCs. EGF withdrawal and MEK inhibition led to a significant increase in the proportion of neuronally committed cells and a corresponding decrease in uncommitted NSCs in both WT and p66KO NSCs, indicating neuronal differentiation. Across all conditions, we found no significant differences between WT and p66KO NSCs in the percentage of live neuronally committed or unknown identity cells. We did, however, observe a significantly higher percentage of live undifferentiated p66KO NSCs following MEK inhibition, which could suggest either a bias in their differentiation or their fate-specific survival.

To assess whether cell identity influenced the survival of p66KO cells, we analyzed the proportion of dead cells within each identity category, measured as the percentage of cells with condensed nuclei (Fig. 7C, D). We found that both uncommitted NSCs and neuronally committed cells in the p66KO population were resistant to cell death induced by EGF withdrawal and MEK inhibition. This indicates that the protective effect of p66Shc deletion to EGFR-ERK pathway inhibition spans different stages of differentiation and is not limited to a specific cell identity.

To further explore the implications of this broad resistance, we investigated the long-term effects of MEK inhibition on the NSCs by extending the treatment duration to 7 days. Though WT NSCs did not survive the treatment, p66KO NSCs remained attached and formed neurite-like projections (Fig. 7E). IF analysis confirmed that these projections were positive for βIII-tubulin, a marker of immature neurons (Fig. 7F). Quantification of the IF data showed a significant increase in βIII-tubulin expression, accompanied by a corresponding loss of Nestin, following 7 days of MEK inhibition (Fig. 7G). These findings indicate that prolonged MEK inhibition drives neuronal differentiation in p66KO cells, reinforcing the conclusion that their resistance to apoptosis is coupled with their ability to progress through differentiation.

In summary, these results indicate that the resistance of p66KO NSCs to MEK inhibition is not driven by differences in initial cell identity or differentiation bias but reflects a broader protective effect that spans both uncommitted and neuronally committed cells. This enhanced resistance allows p66KO NSCs to evade MEK inhibition-induced apoptosis, enabling them to progress through differentiation and develop into neurons.

## Discussion

**Fig. 8:**
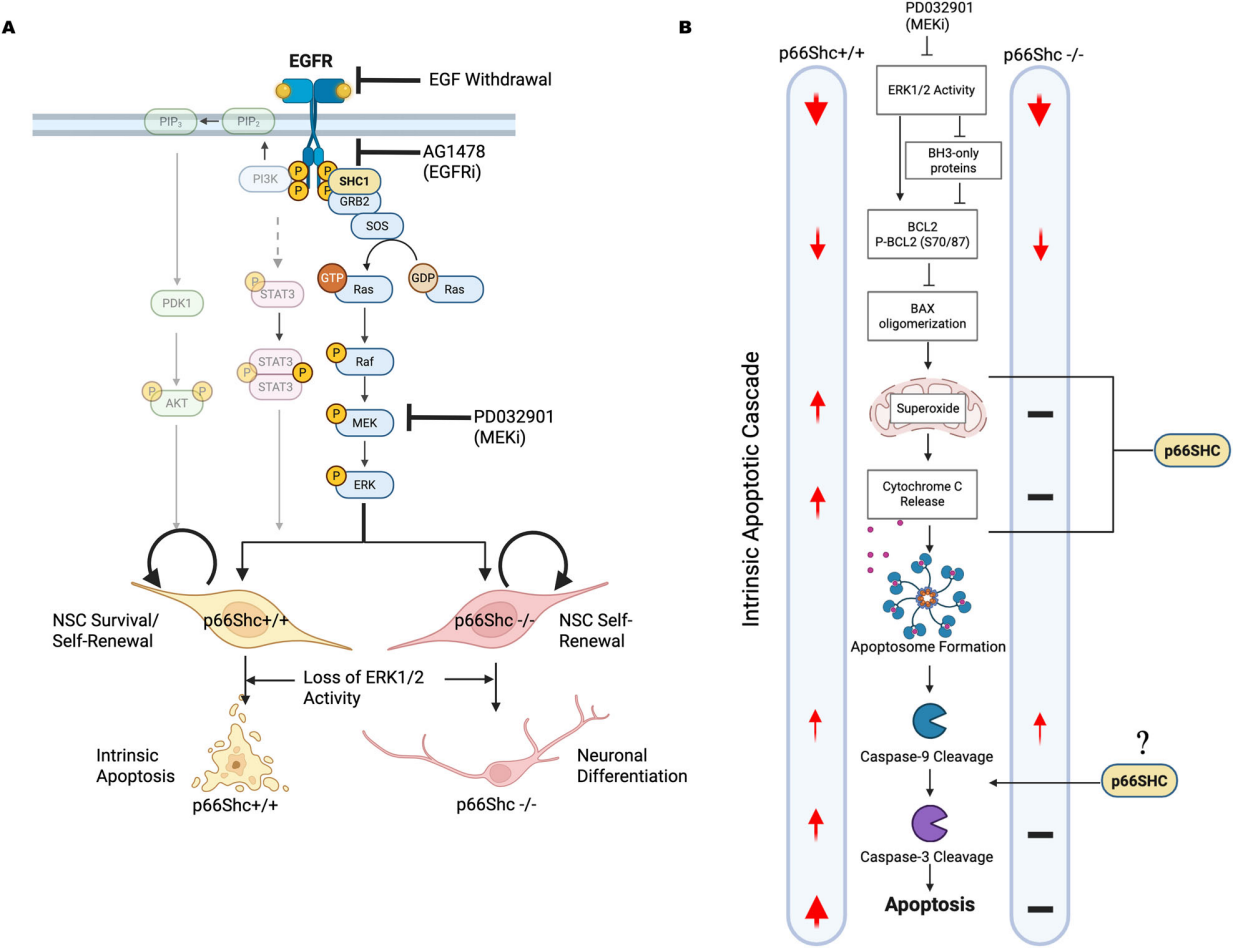
Summary of findings and proposed model positioning p66Shc within the intrinsic apoptotic cascade following MEK inhibition in neural stem cells. **A** Summary of key experimental treatments and findings interrogating EGFR-ERK-mediated survival signalling in wild-type (WT; p66Shc+/+) and knockout (KO; p66Shc−/−) neural stem cells (NSCs). EGF withdrawal, EGFR inhibition (AG1478), and MEK inhibition (PD0325901) each induced apoptosis in WT NSCs but not in p66KO NSCs. These results identify ERK as the critical downstream effector of EGFR-mediated survival in NSCs. Notably, loss of ERK activity triggers apoptosis only in the presence of p66Shc, whereas NSCs lacking p66Shc evade cell death and instead proceed to neuronal differentiation. Created in BioRender. Lab, B. (2025) https://BioRender.com/yvuvlyb. **B** Schematic model of the intrinsic apoptotic cascade in response to MEK inhibition, highlighting differential pathway activation in WT (p66Shc+/+) and p66KO (p66Shc−/−) NSCs, and the proposed positioning of p66Shc within this cascade. Assessed apoptotic events are denoted by adjacent red arrows, with arrow size representing relative magnitude of change and “–” indicating limited or no significant change. Compared to WT NSCs, p66KO NSCs exhibit attenuated mitochondrial ROS production, reduced cytochrome c release, and impaired caspase-3 activation, despite equivalent ERK inhibition and comparable changes to BCL2 expression and phosphorylation. These differences position p66Shc downstream of ERK, likely at the level of the mitochondrion, and potentially also between caspase-9 activation and caspase-3 cleavage. The absence of p66Shc uncouples early apoptotic signalling from downstream execution, thereby conferring resistance to apoptosis induced by EGFR-ERK pathway disruption. Created in BioRender. Lab, B. (2025) https://BioRender.com/7sd195f.

This study reveals that *p66Shc* deletion from NSCs confers resistance to apoptosis induced by EGF withdrawal, EGFR inhibition, and MEK inhibition, primarily through mechanisms independent of compensatory ERK or PI3K-AKT signaling. Specifically, we demonstrate that deleting *p66Shc* enables NSCs to evade apoptosis triggered by ERK signaling axis disruptions while preserving functional apoptotic responses to general stressors like staurosporine. Notably, this resistance to apoptosis was not mediated by changes in cell identity or differentiation capacity but instead reflected a broader intrinsic resistance to ERK pathway perturbation. Furthermore, the loss of p66Shc selectively attenuated the apoptotic response without initiating compensatory cell death mechanisms, such as necrosis, often observed when the apoptotic cascade’s downstream components are inhibited [49–51]. Instead, the p66KO NSCs remained viable, which allowed the NSCs to differentiate toward neuronal lineages despite sustained MEK inhibition. Figure 8 provides a schematic summary of our key experimental findings and proposes a model positioning p66Shc within the intrinsic apoptotic cascade of NSCs following MEK inhibition.

EGF is critical for NSC survival, and its deprivation often induces intrinsic apoptosis [52–54]. While EGF survival signalling in NSCs has been less studied, it is well-characterized in cancers [17]. Notably, half of all glioblastomas multiforme (GBMs), which share many characteristics with NSCs [55–57], overexpress EGFR or have activating mutations with the EGFR coding sequence [58, 59]. In these contexts, EGFR survival signaling is primarily mediated by the STAT3, AKT, and ERK1/2 pathways, highlighting the pathway’s importance across different cell types [17]. SHC1 links EGFR to RAS activation, playing a critical role in amplifying downstream pathways like ERK1/2 and, consequently, is crucial for RTK-mediated survival signaling [22].

Genetic studies conducted in vivo have highlighted that alterations in SHC1 function within NSCs can lead to significant disruptions in brain development. The two existing in vivo studies investigating SHC1 disruption in mouse brains consistently observed microcephaly, yet intriguingly reported different underlying mechanisms for this abnormal brain development. McFarland et al. found that mutations in SHC1 tyrosine phosphorylation sites caused microcephaly due to increased NSC apoptosis [32]. At the same time, Ponti et al. reported that conditional SHC1 deletion led to microcephaly from impaired NSC proliferation without apoptosis [31]. Our findings that p66Shc mediates EGFR-ERK inhibition-induced apoptosis reconcile these differences. Total SHC1 knockout impairs RTK-ERK signaling and p66Shc-mediated apoptosis, whereas SHC1 inactivation affects RTK signaling but not p66Shc’s pro-apoptotic function, which operates independently of tyrosine phosphorylation. This may explain why the absence of p66Shc leads to proliferation loss without inducing apoptosis and why the inactivation of EGFR-SHC-ERK signaling in the presence of p66Shc induces NSC apoptosis.

Although p66Shc is known to promote apoptosis in various contexts, its precise mechanisms remain controversial. Proposed mechanisms include promoting ROS production [37, 60, 61], collapsing mitochondrial transmembrane potential [36], disrupting mitochondrial dynamics [62], disrupting calcium homeostasis [63], interacting with p53 to enhance stress-induced apoptosis [39], and inhibiting survival pathways like Ras/ERK and PI3K/AKT [64–66]. Particularly, p66Shc’s direct influence on receptor tyrosine kinase signaling—a key component of cell survival pathways—complicates isolating its specific contribution to apoptosis.

In this study, we’ve attempted to disentangle p66Shc’s signaling role from its pro-apoptotic effects by inhibiting EGFR, its primary receptor and a significant contributor to NSC survival in vitro. It is often theorized that p66Shc promotes apoptosis by monopolizing Shc binding sites, thus preventing other isoforms like p52 and p46Shc from activating EGFR pro-survival signals [33, 67]. However, our findings—that loss of p66Shc enhances survival, specifically under EGFR and MEK inhibition—contradict this model. We observed that pharmacologic inhibition of EGFR and MEK significantly reduces ERK signalling in WT and p66KO NSCs but induces apoptosis only in WT NSCs. These results suggest that p66Shc exerts its pro-apoptotic effect through a mechanism independent of Shc’s influence on EGFR and ERK signaling.

An alternative explanation for p66Shc’s pro-apoptotic function is its role as a mitochondrial redox modulator and its contribution to oxidative stress. Consistent with theory, we observed that MEKi-treated WT NSCs exhibited higher ROS levels than p66KO NSCs; however, antioxidant treatment did not rescue cell survival. One limitation of this approach is that p66Shc localizes to the intermembrane space, whereas MitoTEMPO accumulates in the matrix [68], potentially limiting its ability to neutralize ROS at the relevant site of action. Our finding that the p66KO NSCs displayed moderate resistance to rotenone, a complex I inhibitor, provides partial support for the role of p66Shc in sensitizing cells to oxidative stress, although its deletion did not confer resistance to antimycin A, a complex III inhibitor. Importantly, their enhanced resistance to MEK inhibition was substantially more pronounced than to either mitochondrial stressor, indicating that p66Shc more specifically contributes to NSC sensitivity in the context of ERK pathway disruption than in response to direct oxidative stress. This raised the possibility that p66Shc’s redox-modulating function, while relevant, may be governed by additional context-specific mechanisms beyond ROS generation alone.

Indeed, the redox-modulating function of p66Shc may be influenced by upstream regulatory events. Phosphorylation of p66Shc at serine 36 is a well-established mechanism for controlling its pro-apoptotic activity, particularly in response to oxidative stress [37, 38]. Although we were unable to reliably detect phosphorylated p66Shc in NSCs by immunoblotting—despite using multiple phospho-specific antibodies and immunoprecipitation—this limitation does not preclude a role for serine 36 phosphorylation in the context of EGFR or MEK inhibition. Future studies using phospho-deficient or phospho-mimetic mutants will be important to clarify whether this post-translational modification contributes to p66Shc activation in ERK inhibition–mediated apoptosis.

Our findings suggest that p66Shc is positioned downstream of ERK, where ERK acts to inhibit p66Shc-mediated apoptosis; however, the specific regulatory mechanism remains uncertain. Evidence indicates that p66Shc and ERK1 may form a complex, with p66Shc’s CH2 domain potentially functioning as an ERK docking site [69]. This interaction could allow ERK to inhibit p66Shc’s pro-apoptotic activity by direct phosphorylation.

MEK-inhibition induced the loss of procaspase-9 in both WT and p66Shc KO NSCs; however, cleavage of caspase-3 occurred only in WT cells. This may reflect a relief of ERK-mediated phosphorylation of caspase-9—particularly at Thr125—which suppresses its activation under survival conditions [70]. MEK inhibition may permit partial caspase-9 activation in both genotypes, even without cytochrome c release. However, insufficient cytochrome c-mediated apoptosome formation may limit the amplification of caspase-9 activity required for caspase-3 cleavage. Alternatively, caspase-9 loss in p66Shc KO cells may result from cleavage at non-activating sites. Though this finding is peripheral to the focus of the study, it warrants further investigation.

This study reveals that p66Shc is critical in mediating the apoptosis of NSCs under disrupted growth factor signaling. The discovery that p66Shc deletion confers resistance to apoptosis induced by EGF withdrawal, EGFR inhibition, and MEK inhibition—while leaving responses to general stressors intact—indicates that p66Shc functions as a gatekeeper for ERK-regulated apoptotic pathways. These findings challenge the conventional view that p66Shc promotes apoptosis primarily by inhibiting RTK signaling or ROS production. Instead, our data position p66Shc as a context-dependent effector of cell death, specifically in response to perturbations in EGFR-ERK signalling.

The ability of p66KO NSCs to evade apoptosis and continue differentiation under sustained MEK inhibition highlights the potential of targeting p66Shc to preserve NSC viability in neurodegenerative diseases or brain injuries where growth factor signaling may be compromised. Conversely, given that p66Shc is absent in certain cancers [66, 71, 72], its expression could serve as a prognostic marker for sensitivity to EGFR- or MEK-targeted therapies. Therapeutic modulation of p66Shc levels could enhance the efficacy of such treatments by restoring apoptotic sensitivity in otherwise resistant tumors.

One promising approach to modulating p66Shc expression is through histone deacetylase inhibitors, such as vorinostat (SAHA) and trichostatin A. These agents reverse epigenetic silencing of *SHC1* by increasing histone acetylation at its promoter, resulting in elevated p66Shc expression and mitochondrial ROS production [73–75]. These studies, together with our own findings, raise the possibility that HDAC inhibition could be employed to reactivate p66Shc-driven apoptotic pathways in NSC-like cancers or other cells that have silenced *SHC1*, thereby improving therapeutic responses to EGFR or MEK inhibition.

## Supplementary information

Supplementary Figure S1

Supplementary Figure S2

Supplementary Figure S3

Supplementary Figure S4

Supplementary Figure Captions

Supplementary Table

Original Data

## Data Availability

Full-length blots and qPCR data are presented in the supplementary Original Data, and all other data are available upon request.
