# Supplementary figures and images for: p66Shc deletion confers apoptotic resistance to loss of EGFR-ERK signalling in neural stem cells
Source: Cell Death Dis. 2025 Jul 1;16(1):479. doi: 10.1038/s41419-025-07778-8 (PMC12217751; doi:10.1038/s41419-025-07778-8)

**A**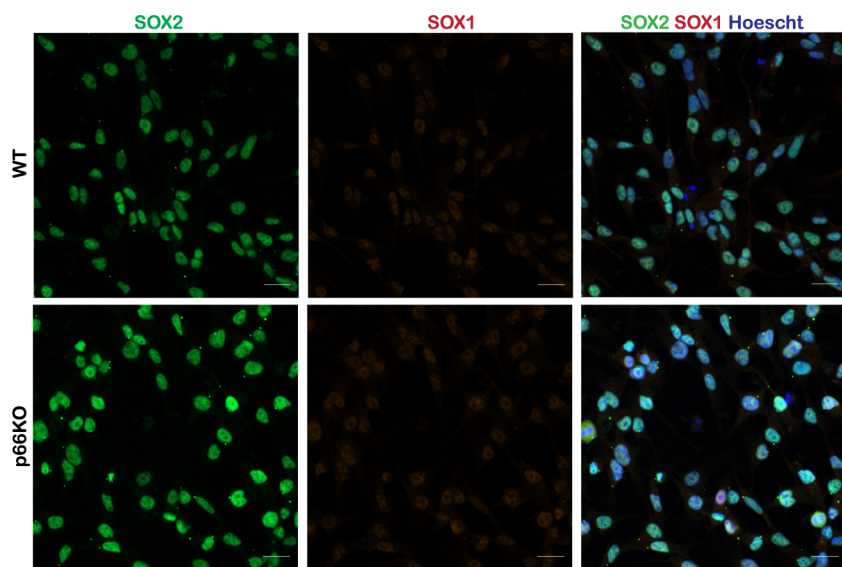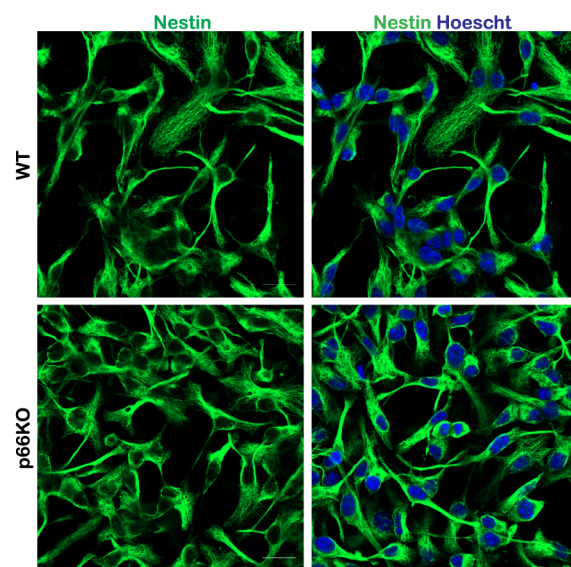**B**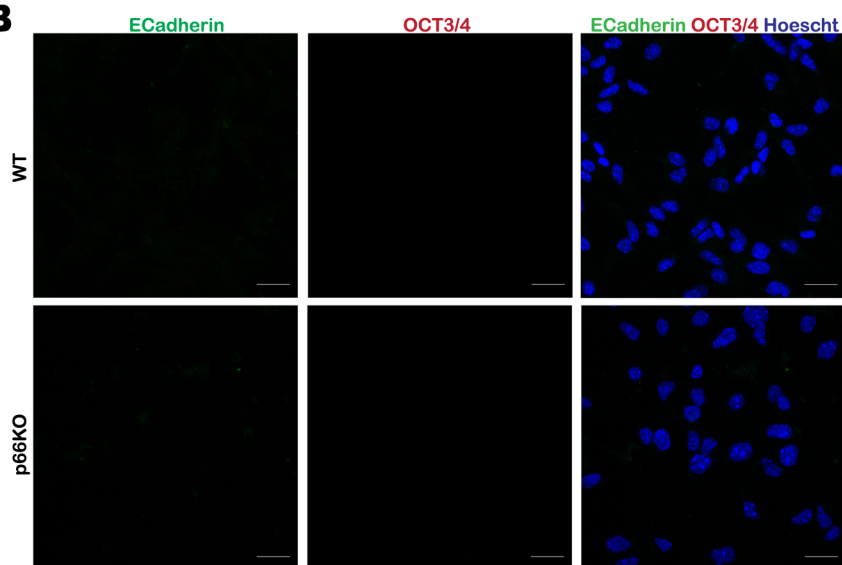

Supplement: Supplementary file 1 — Supplementary Figure S1 [file 41419_2025_7778_MOESM1_ESM.pdf]

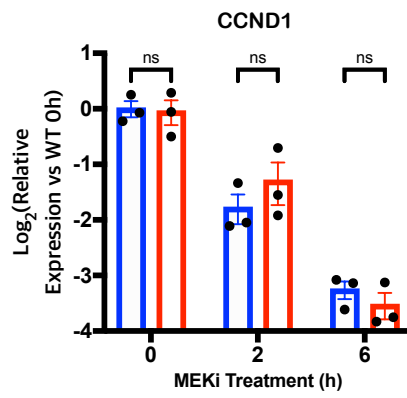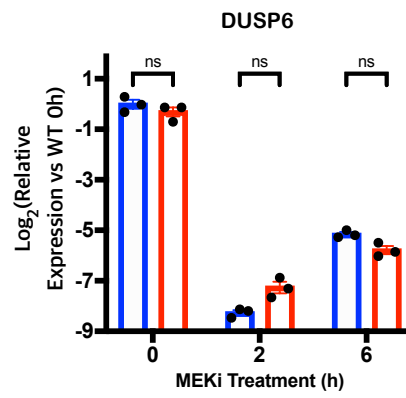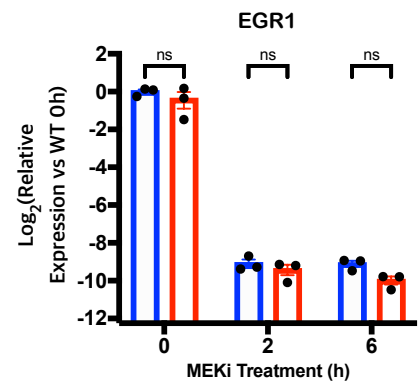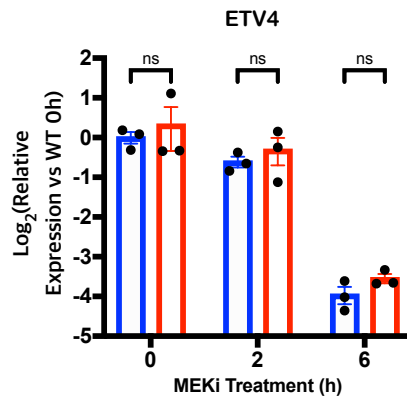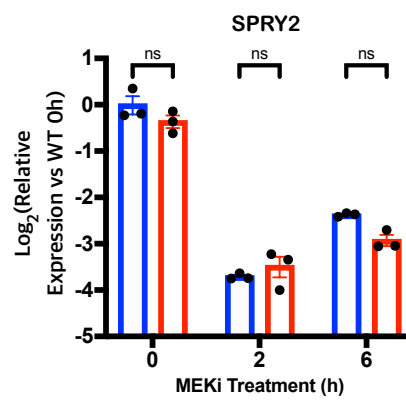

WT  
KO

Supplement: Supplementary file 2 — Supplementary Figure S2 [file 41419_2025_7778_MOESM2_ESM.pdf]

**A**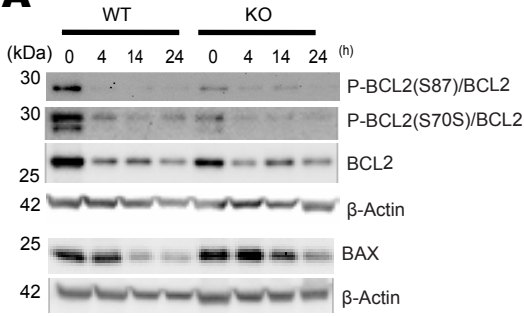**B**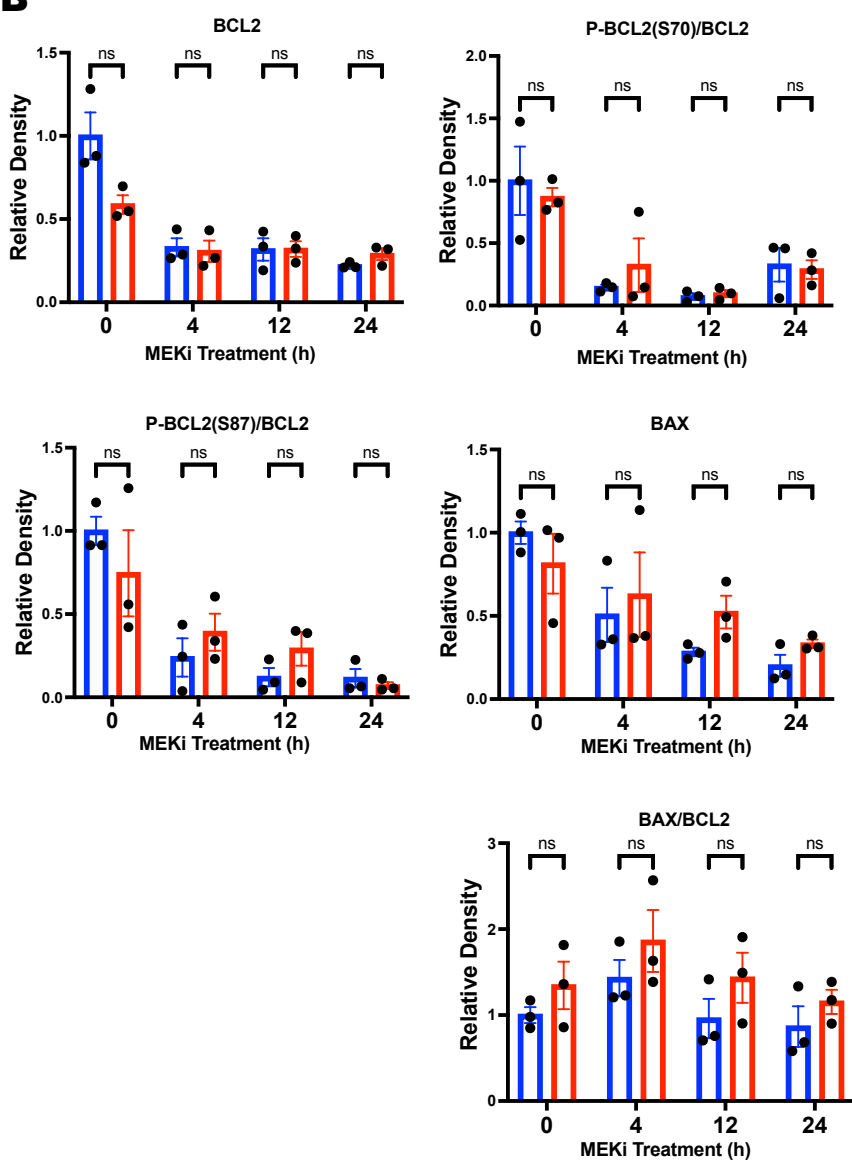

Supplement: Supplementary file 3 — Supplementary Figure S3 [file 41419_2025_7778_MOESM3_ESM.pdf]

**A**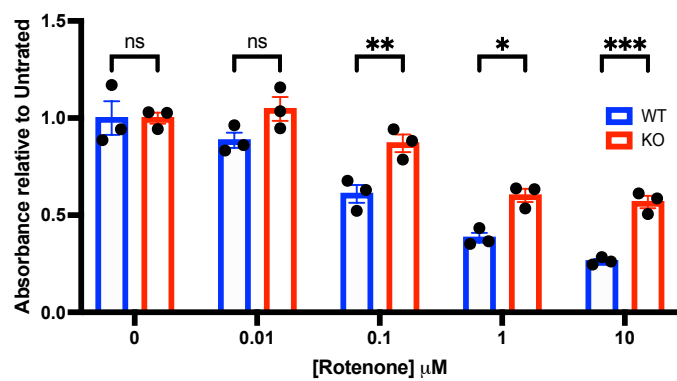**B**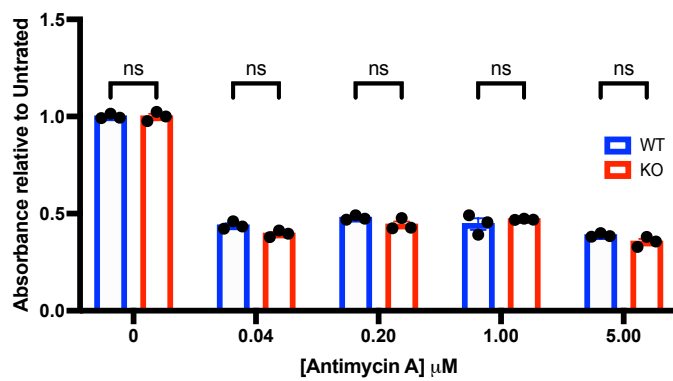**C**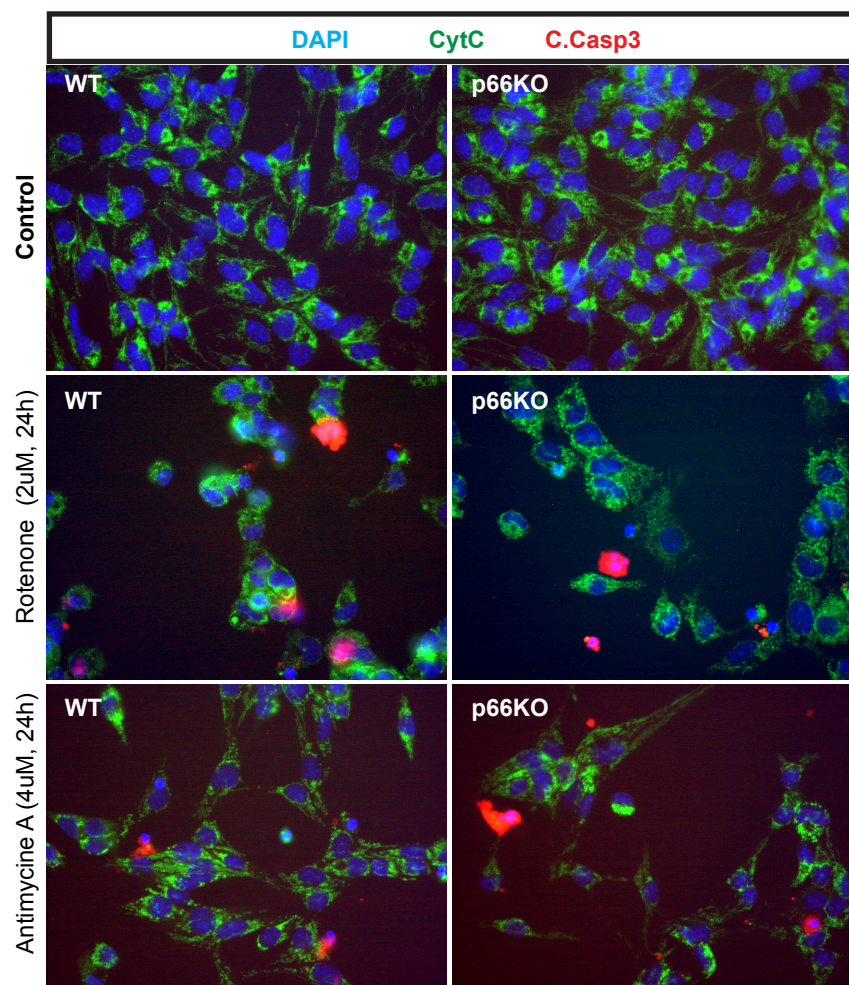**D**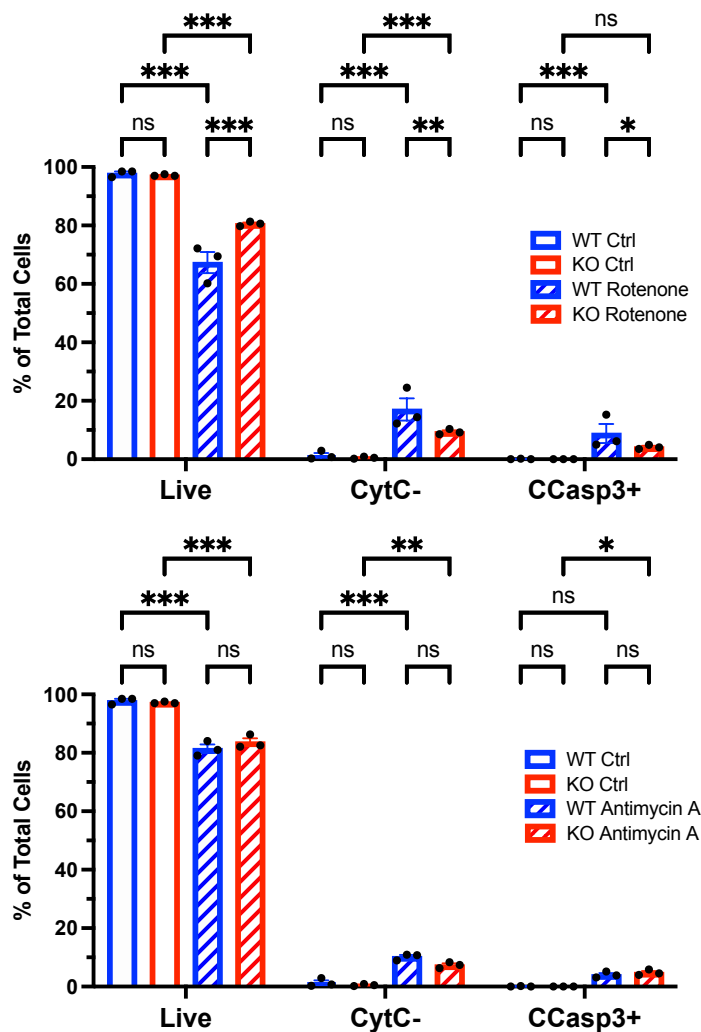

Supplement: Supplementary file 4 — Supplementary Figure S4 [file 41419_2025_7778_MOESM4_ESM.pdf]
