# Supplementary material for: p66Shc deletion confers apoptotic resistance to loss of EGFR-ERK signalling in neural stem cells
Source: Cell Death Dis. 2025 Jul 1;16(1):479. doi: 10.1038/s41419-025-07778-8 (PMC12217751; doi:10.1038/s41419-025-07778-8)
Supplement: Supplementary file 5 — Supplementary Figure Captions [file 41419_2025_7778_MOESM5_ESM.docx]

**Figure S1: p66KO NSCs express the NSC identity markers SOX2 and Nestin and lack embryonic identity markers OCT3/4 and E-cadherin**Representative IF images of WT and p66KO NSCs, stained for NSC identity markers SOX2 and Nestin and the neural ectoderm marker SOX1 (A), and stained for embryonic stem cell markers E-cadherin and OCT3/4 (B). Scale=10 μm.

**Figure S2.** **Quantification of ERK target gene expression following MEK inhibition in WT and p66KO NSCs.**
WT and p66KO NSCs were treated with 1 μM PD0325901 for 0, 2, or 6 hours. *CCND1*, *DUSP6*, *EGR1*, *ETV4*, and *SPRY2* transcript abundance were measured by qPCR and normalized to GAPDH and Actin. Displayed values are relative to untreated WT NSCs. ERK inhibition significantly suppressed the expression of all five ERK-responsive genes in both genotypes, confirming the effective inhibition of ERK signaling.

**Figure S3: MEK inhibition similarly disrupts BCL2 signaling in WT and p66KO NSCs**
Western blot analysis of total BCL2, BAX, and BCL2 phosphorylation at ERK-regulated sites S70 and S87 in WT and p66KO NSCs treated with 1 μM PD0325901 over a 24 h time course. Representative blots A and densitometric quantification B are shown. MEK inhibition led to a comparable reduction in BCL2 phosphorylation at both sites in WT and p66KO cells, with no significant differences in total BCL2, BAX, or BAX/BCL2 ratios across genotypes. Data are presented as mean ± SEM; n = 3 independent experiments. Statistical analysis was performed using two-way ANOVA: ns, p≥0.05; *, p<0.05; **, p<0.01; ***p<0.001.

**Figure S4: p66Shc deletion confers partial resistance to mitochondrial ROS-inducing agents**
**A:** Viability of WT and p66KO NSCs following 24-hour treatment with increasing concentrations of rotenone (0.01–10 μM), assessed by MTT assay. Absorbance values are normalized to untreated controls; mean ± SEM, n = 3 independent experiments.
**B:** Viability of WT and p66KO NSCs following 24-hour treatment with increasing concentrations of antimycin A (0.04–5 μM), assessed by MTT assay. Absorbance values are normalized to untreated controls; mean ± SEM, n = 3 independent experiments.
**C:** Representative immunofluorescence images of WT and p66KO NSCs treated with rotenone (2 μM, 24 h) or antimycin A (4 μM, 24 h), stained for cleaved caspase-3 and cytochrome c. Scale = 50 μm.
Statistics were obtained using two-way ANOVA: ns, p ≥ 0.05; *, p < 0.05; **, p < 0.01; ***p < 0.001.
**D:** Quantification of apoptotic markers by immunofluorescence following treatment with rotenone (2 μM, 24 h) or antimycin A (4 μM, 24 h). Data are shown as the percentage of cells positive for cleaved caspase-3 (CCasp3⁺) and/or negative for cytochrome c (CytC⁻); mean ± SEM, n = 3 independent experiments.
Statistics were obtained using two-way ANOVA: ns, p ≥ 0.05; *, p < 0.05; **, p < 0.01; ***p < 0.001.
