# Supplementary material for: p66Shc deletion confers apoptotic resistance to loss of EGFR-ERK signalling in neural stem cells
Source: Cell Death Dis. 2025 Jul 1;16(1):479. doi: 10.1038/s41419-025-07778-8 (PMC12217751; doi:10.1038/s41419-025-07778-8)
Supplement: Supplementary file 6 — Supplementary Table [file 41419_2025_7778_MOESM6_ESM.pdf]

Table 1. Primary antibodies used for immunofluorescence.

Antibody	Species	Concentration	Catalog number
OCT3/4	Mouse	1:200	Santa Cruz, sc-5279
SOX2	Rabbit	1:400	Cell Signaling, 3579
Nestin	Mouse	1:400	Abcam, ab6142
Doublecortin	Rabbit	1:400	Abcam, ab18723
BIII-Tubulin	Mouse	1:400	Abcam, ab78078
BIII-Tubulin	Rabbit	1:400	Abcam, ab18207
GFAP	Rabbit	1:400	Abcam, ab7260
Cleaved Caspase 3	Rabbit	1:400	Cell Signaling, 9664
Cytochrome C	Mouse	1:400	Invitrogen, 33-8200
E-Cadherin	Rabbit	1:200	Cell Signaling, 3195

Table 2. Primary antibodies used for immunoblotting.

Antibody	Species	Concentration	Catalog number
SHCA	Mouse	1:1000	BD Biosciences, 610879
P-SHC (Y317)	Rabbit	1:1000	Cell Signaling, 2431
P-SHC (Y239/240)	Rabbit	1:1000	Cell Signaling, 2434
P-ERK (P-p44/42 MAPK (T202/Y204)	Rabbit	1:1000	Cell Signaling, 9101
ERK (p44/42 MAPK)	Rabbit	1:1000	Cell Signaling, 4695
P-STAT3 Y705	Rabbit	1:1000	Cell Signaling, 9145
P-STAT3 S727	Rabbit	1:1000	Cell Signaling, 9134
STAT3	Mouse	1:1000	Santa Cruz, sc-8019
P-AKT (S473)	Rabbit	1:1000	Cell Signaling, 9271
AKT	Mouse	1:2000	Cell Signaling, 2920
Actin-HRP conjugate	Rabbit	1:10000	Cell Signaling, 5125
Cleaved Caspase-3	Rabbit	1:1000	Cell Signaling, 9664
Caspase-3	Rabbit	1:2000	Cell Signaling, 9662
Caspase-9	Rabbit	1:1000	Cell Signaling, 9504
BCL2	Rabbit	1:500	SantaCruz, SC7382
P-BCL2(S70)	Rabbit	1:500	ThermoFisher, 500-11574
P-BCL2(S87)	Rabbit	1:500	Sigma, sab5700577
BAX	Mouse	1:500	Santa Cruz, sc-7480

Table 3. Primers used for qPCR to assess ERK-responsive transcripts.

Antibody	Catalog number
DUSP6	F: ATAGATACGCTCAGACCCGTG<br>R: ATCAGCAGAAGCCGTTCGTT
EGR1	F: AGCGAACAACCCTATGAGCACC<br>R: ATGGGAGGCAACCGAGTCGTTT
SPRY2	F: TCCAAGAGATGCCCTTACCCA<br>R: GCAGACCGTGGAGTCTTTCA
ETV4	F: CACAGACTTCGCCTACGACTCA<br>R: GCAGACATCATCTGGGAATGGTC
CCND1	F: GCAGAAGGAGATTGTGCCATCC<br>R: AGGAAGCGGTCCAGGTAGTTCA
GAPDH	F: GAAGGGCTCATGACCACAGTCC<br>R: TCATACTTGGCAGGTTTCTCCAGGC
ACTIN	F: GCTGTATTCCCCTCCATCGTG<br>R: CACGGTTGGCCTTAGGGTTCAG
