# Supplementary material for: p66Shc deletion confers apoptotic resistance to loss of EGFR-ERK signalling in neural stem cells
Source: Cell Death Dis. 2025 Jul 1;16(1):479. doi: 10.1038/s41419-025-07778-8 (PMC12217751; doi:10.1038/s41419-025-07778-8)
Supplement: Supplementary file 7 — Original Data [file 41419_2025_7778_MOESM7_ESM.pdf]

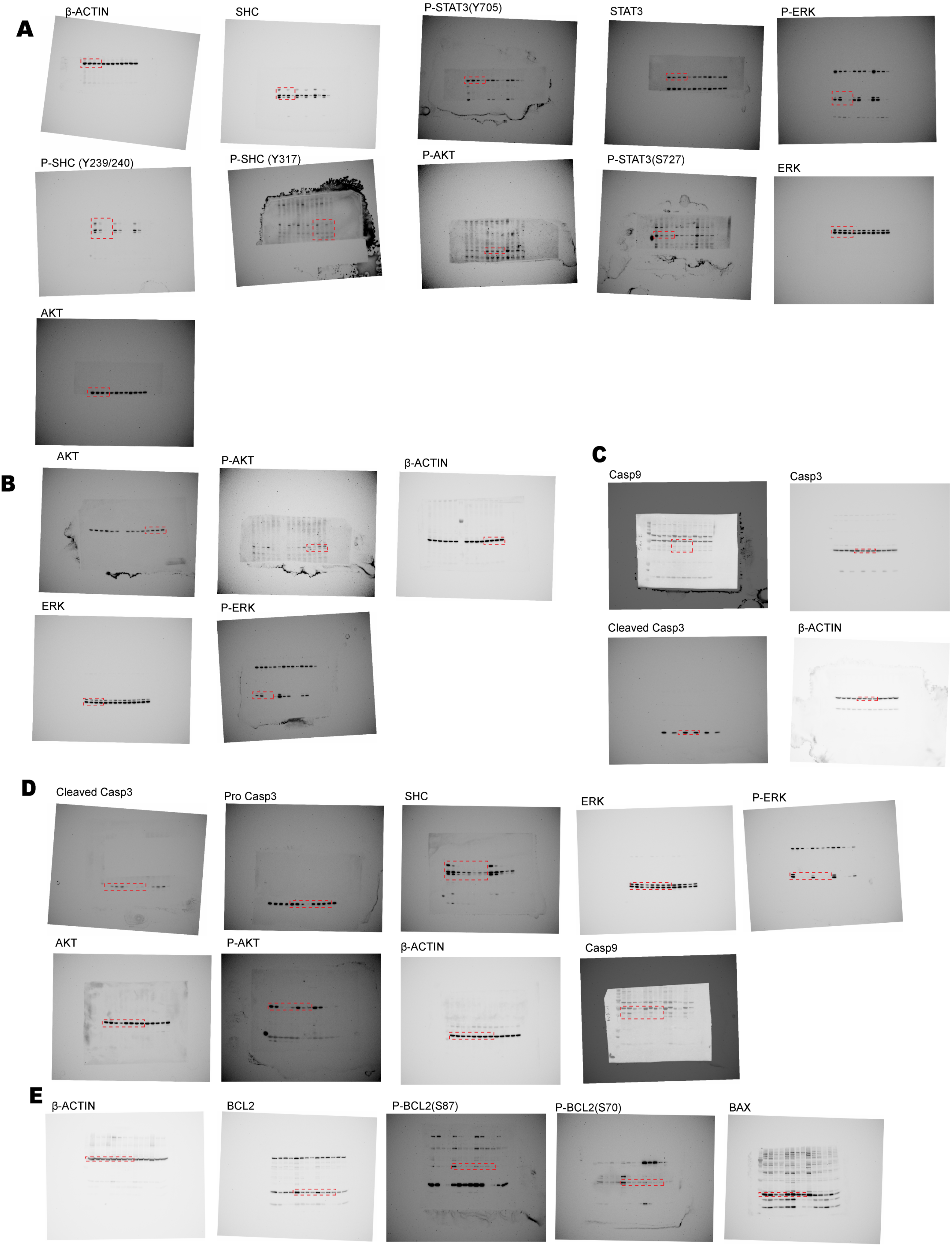
A
β-ACTIN
SHC
P-STAT3(Y705)
STAT3
P-ERK
P-SHC (Y239/240)
P-SHC (Y317)
P-AKT
P-STAT3(S727)
ERK
AKT
B
AKT
P-AKT
β-ACTIN
ERK
P-ERK
C
Casp9
Casp3
Cleaved Casp3
β-ACTIN
D
Cleaved Casp3
Pro Casp3
SHC
ERK
P-ERK
AKT
P-AKT
β-ACTIN
Casp9
E
β-ACTIN
BCL2
P-BCL2(S87)
P-BCL2(S70)
BAX

	Target	Sample	Mean Cq	Mean Efficiency Corrected Cq	Normalized Expression	Relative Normalized Expression	Fold Change	Target Regulation	Average	malized to WT 0
2	Actin	KO 0 N1	19.11	19.72				No change		
3	Actin	KO 0 N2	19.73	20.37				No change		
4	Actin	KO 0 N3	19.32	19.94				No change		
5	Actin	KO 2 N1	18.82	19.42				No change		
6	Actin	KO 2 N2	18.23	18.81				No change		
7	Actin	KO 2 N3	17.91	18.49				No change		
8	Actin	KO 6 N1	18.95	19.56				No change		
9	Actin	KO 6 N2	18.41	19.00				No change		
10	Actin	KO 6 N3	18.17	18.75				No change		
11	Actin	WT 0 N1	18.81	19.42				No change		
12	Actin	WT 0 N2	18.58	19.18				No change		
13	Actin	WT 0 N3	18.64	19.24				No change		
14	Actin	WT 2 N1	19.11	19.73				No change		
15	Actin	WT 2 N2	19.20	19.81				No change		
16	Actin	WT 2 N3	19.41	20.04				No change		
17	Actin	WT 6 N1	18.89	19.50				No change		
18	Actin	WT 6 N2	19.10	19.71				No change		
19	Actin	WT 6 N3	19.60	20.23				No change		
20	CCND1	KO 0 N1	21.07	23.80	0.04684	5.97692	5.97692	Up regulated		0.7072027
21	CCND1	KO 0 N2	21.06	23.79	0.08082	10.31445	10.31445	Up regulated		1.2204293
22	CCND1	KO 0 N3	21.12	23.86	0.06376	8.13699	8.13699	Up regulated		0.9627873
23	CCND1	KO 2 N1	21.50	24.28	0.04060	5.18143	5.18143	Up regulated		0.6130781
24	CCND1	KO 2 N2	21.53	24.32	0.02260	2.88461	2.88461	No change		0.3413137
25	CCND1	KO 2 N3	21.57	24.36	0.01752	2.23557	2.23557	No change		0.264518
26	CCND1	KO 6 N1	24.08	27.20	0.00466	0.59406	-1.68332	No change		0.0702911
27	CCND1	KO 6 N2	22.88	25.85	0.00759	0.96848	-1.03255	No change		0.1145925
28	CCND1	KO 6 N3	23.56	26.61	0.00492	0.62765	-1.59325	No change		0.0742649
29	CCND1	WT 0 N1	20.41	23.05	0.07894	10.07404	10.07404	Up regulate	8.45150	1.1919826
30	CCND1	WT 0 N2	20.61	23.28	0.06301	8.04099	8.04099	Up regulated		0.9514277
31	CCND1	WT 0 N3	20.68	23.36	0.05673	7.23947	7.23947	Up regulated		0.8565897
32	CCND1	WT 2 N1	22.12	24.98	0.02623	3.34727	3.34727	No change		0.3960561
33	CCND1	WT 2 N2	23.06	26.05	0.01535	1.95916	1.95916	No change		0.2318126
34	CCND1	WT 2 N3	22.77	25.72	0.01603	2.04520	2.04520	No change		0.241993
35	CCND1	WT 6 N1	23.42	26.45	0.00784	1.00000	1.00000	No change		0.1183222
36	CCND1	WT 6 N2	23.62	26.68	0.00752	0.95931	-1.04242	No change		0.1135072
37	CCND1	WT 6 N3	24.47	27.64	0.00541	0.69056	-1.44809	No change		0.0817092
38	DUSP6	KO 0 N1	21.97	21.70	0.20058	23.72054	23.72054	Up regulated		0.6134506
39	DUSP6	KO 0 N2	22.18	21.91	0.29787	35.22740	35.22740	Up regulated		0.911036
40	DUSP6	KO 0 N3	21.91	21.64	0.29786	35.22594	35.22594	Up regulated		0.9109982
41	DUSP6	KO 2 N1	28.50	28.15	0.00278	0.32865	-3.04272	No change		0.0084995
42	DUSP6	KO 2 N2	28.12	27.77	0.00206	0.24375	-4.10254	Down regulated		0.0063038
43	DUSP6	KO 2 N3	28.15	27.80	0.00162	0.19100	-5.23567	Down regulated		0.0049395
44	DUSP6	KO 6 N1	26.89	26.56	0.00725	0.85778	-1.16580	No change		0.0221835
45	DUSP6	KO 6 N2	26.60	26.27	0.00565	0.66876	-1.49531	No change		0.0172951
46	DUSP6	KO 6 N3	26.91	26.58	0.00502	0.59318	-1.68582	No change		0.0153406
47	DUSP6	WT 0 N1	21.29	21.03	0.32156	38.02871	38.02871	Up regulate	38.66740	0.9834824
48	DUSP6	WT 0 N2	20.87	20.62	0.39858	47.13716	47.13716	Up regulated		1.2190413
49	DUSP6	WT 0 N3	21.42	21.16	0.26074	30.83634	30.83634	Up regulated		0.7974763
50	DUSP6	WT 2 N1	29.90	29.54	0.00112	0.13196	-7.57817	Down regulated		0.0034126

	A	B	C	D	E	F	G	H	I	J	K	L
51		DUSP6	WT 2 N2	30.48	30.11	0.00092	0.10930	-9.14945	Down regulated		0.0028266	
52		DUSP6	WT 2 N3	29.87	29.50	0.00117	0.13795	-7.24918	Down regulated		0.0035675	
53		DUSP6	WT 6 N1	26.67	26.34	0.00846	1.00000	1.00000	No change		0.0258616	
54		DUSP6	WT 6 N2	26.56	26.24	0.01024	1.21117	1.21117	No change		0.0313228	
55		DUSP6	WT 6 N3	27.25	26.92	0.00891	1.05318	1.05318	No change		0.0272369	
56		EGR1	KO 0 N1	22.23	26.49	0.00723	255.35430	255.35430	Up regulated		0.3578769	
57		EGR1	KO 0 N2	21.50	25.62	0.02269	801.24319	801.24319	Up regulated		1.1229355	
58		EGR1	KO 0 N3	21.71	25.87	0.01585	559.87758	559.87758	Up regulated		0.7846636	
59		EGR1	KO 2 N1	28.95	34.49	0.00003	1.21374	1.21374	No change		0.001701	
60		EGR1	KO 2 N2	28.21	33.62	0.00004	1.26782	1.26782	No change		0.0017768	
61		EGR1	KO 2 N3	28.74	34.25	0.00002	0.65374	-1.52965	No change		0.0009162	
62		EGR1	KO 6 N1	29.27	34.87	0.00002	0.80509	-1.24210	No change		0.0011283	
63		EGR1	KO 6 N2	28.72	34.22	0.00002	0.81046	-1.23386	No change		0.0011359	
64		EGR1	KO 6 N3	29.41	35.05	0.00001	0.49990	-2.00038	No change		0.0007006	
65		EGR1	WT 0 N1	20.88	24.88	0.02223	785.09878	785.09878	Up regulatec	713.52557	1.1003092	
66		EGR1	WT 0 N2	21.13	25.18	0.01687	595.81891	595.81891	Up regulated		0.8350351	
67		EGR1	WT 0 N3	20.78	24.76	0.02151	759.65904	759.65904	Up regulated		1.0646557	
68		EGR1	WT 2 N1	28.58	34.06	0.00005	1.71535	1.71535	No change		0.002404	
69		EGR1	WT 2 N2	29.41	35.04	0.00003	1.06988	1.06988	No change		0.0014994	
70		EGR1	WT 2 N3	29.09	34.67	0.00003	1.14815	1.14815	No change		0.0016091	
71		EGR1	WT 6 N1	29.01	34.56	0.00003	1.00000	1.00000	No change		0.0014015	
72		EGR1	WT 6 N2	28.69	34.18	0.00004	1.46441	1.46441	No change		0.0020524	
73		EGR1	WT 6 N3	29.12	34.70	0.00004	1.43261	1.43261	No change		0.0020078	
74		ETV4	KO 0 N1	22.03	25.55	0.01391	16.12411	16.12411	Up regulated		0.78805	
75		ETV4	KO 0 N2	22.69	26.32	0.01404	16.26951	16.26951	Up regulated		0.79516	
76		ETV4	KO 0 N3	21.22	24.60	0.03812	44.18298	44.18298	Up regulated		2.15940	
77		ETV4	KO 2 N1	21.85	25.34	0.01960	22.71720	22.71720	Up regulated		1.11028	
78		ETV4	KO 2 N2	21.49	24.92	0.01487	17.23050	17.23050	Up regulated		0.84212	
79		ETV4	KO 2 N3	21.97	25.48	0.00811	9.39529	9.39529	Up regulated		0.45919	
80		ETV4	KO 6 N1	24.67	28.61	0.00175	2.03325	2.03325	No change		0.09937	
81		ETV4	KO 6 N2	24.41	28.31	0.00138	1.59850	1.59850	No change		0.07813	
82		ETV4	KO 6 N3	24.51	28.42	0.00140	1.62003	1.62003	No change		0.07918	
83		ETV4	WT 0 N1	21.59	25.03	0.02005	23.23582	23.23582	Up regulatec	20.46078	1.13563	
84		ETV4	WT 0 N2	21.93	25.43	0.01420	16.45906	16.45906	Up regulated		0.80442	
85		ETV4	WT 0 N3	21.52	24.96	0.01871	21.68746	21.68746	Up regulated		1.05995	
86		ETV4	WT 2 N1	22.36	25.93	0.01360	15.76127	15.76127	Up regulated		0.77032	
87		ETV4	WT 2 N2	23.02	26.69	0.00986	11.42963	11.42963	Up regulated		0.55861	
88		ETV4	WT 2 N3	22.62	26.24	0.01122	13.00055	13.00055	Up regulated		0.63539	
89		ETV4	WT 6 N1	25.56	29.64	0.00086	1.00000	1.00000	No change		0.04887	
90		ETV4	WT 6 N2	25.06	29.06	0.00144	1.67453	1.67453	No change		0.08184	
91		ETV4	WT 6 N3	25.83	29.95	0.00109	1.26334	1.26334	No change		0.06174	
92		GAPDH	KO 0 N1	19.26	19.04				No change			
93		GAPDH	KO 0 N2	20.19	19.96				No change			
94		GAPDH	KO 0 N3	20.07	19.84				No change			
95		GAPDH	KO 2 N1	20.14	19.90				No change			
96		GAPDH	KO 2 N2	19.11	18.89				No change			
97		GAPDH	KO 2 N3	18.79	18.57				No change			
98		GAPDH	KO 6 N1	19.57	19.34				No change			
99		GAPDH	KO 6 N2	18.83	18.61				No change			
100		GAPDH	KO 6 N3	19.36	19.13				No change			
101		GAPDH	WT 0 N1	19.59	19.36				No change			
102		GAPDH	WT 0 N2	19.63	19.40				No change			

	A	B	C	D	E	F	G	H	I	J	K	L
103		GAPDH	WT 0 N3	19.42	19.20				No change			
104		GAPDH	WT 2 N1	19.96	19.73				No change			
105		GAPDH	WT 2 N2	20.48	20.24				No change			
106		GAPDH	WT 2 N3	19.71	19.48				No change			
107		GAPDH	WT 6 N1	19.64	19.41				No change			
108		GAPDH	WT 6 N2	19.77	19.54				No change			
109		GAPDH	WT 6 N3	20.22	19.99				No change			
110		SPRY2	KO 0 N1	22.26	24.10	0.03792	3.30317	3.30317	No change			
111		SPRY2	KO 0 N2	22.75	24.63	0.04525	3.94212	3.94212	No change			
112		SPRY2	KO 0 N3	22.29	24.14	0.05267	4.58823	4.58823	Up regulated			
113		SPRY2	KO 2 N1	25.04	27.11	0.00572	0.49855	-2.00582	No change			
114		SPRY2	KO 2 N2	24.18	26.18	0.00622	0.54202	-1.84495	No change			
115		SPRY2	KO 2 N3	24.60	26.63	0.00363	0.31652	-3.15938	No change			
116		SPRY2	KO 6 N1	24.25	26.26	0.00892	0.77742	-1.28631	No change			
117		SPRY2	KO 6 N2	23.98	25.97	0.00699	0.60930	-1.64124	No change			
118		SPRY2	KO 6 N3	24.10	26.09	0.00704	0.61324	-1.63068	No change			
119		SPRY2	WT 0 N1	21.38	23.15	0.07406	6.45172	6.45172	Up regulated			
120		SPRY2	WT 0 N2	21.79	23.59	0.05080	4.42502	4.42502	Up regulated			
121		SPRY2	WT 0 N3	21.76	23.55	0.04958	4.31864	4.31864	Up regulated			
122		SPRY2	WT 2 N1	25.47	27.58	0.00433	0.37684	-2.65365	No change			
123		SPRY2	WT 2 N2	25.65	27.77	0.00467	0.40654	-2.45976	No change			
124		SPRY2	WT 2 N3	25.50	27.60	0.00435	0.37867	-2.64080	No change			
125		SPRY2	WT 6 N1	23.92	25.90	0.01148	1.00000	1.00000	No change			
126		SPRY2	WT 6 N2	24.10	26.10	0.01127	0.98214	-1.01819	No change			
127		SPRY2	WT 6 N3	24.59	26.63	0.01088	0.94809	-1.05475	No change			

	A	B	C
1	File Name	ERK transcription targets effect of MEKi on NSCs_2025-03-31 16-05-43_CC008079.pcrd	
2	Created By User	admin	
3	Notes		
4	ID		
5	Run Started	03/31/2025 20:06:31 UTC	
6	Run Ended	03/31/2025 21:43:56 UTC	
7	Sample Vol	10	
8	Lid Temp	95	
9	Protocol File Name	SensiFast SYBR Protocol.prcl	
10	Plate Setup File Name	2025 qPCR NSC ERK activity.pltd	
11	Base Serial Number	CC008079	
12	Optical Head Serial Number	786BR1001	
13	CFX Maestro Version	4.1.2433.1219.	
